# *Vanilla* beyond *Vanilla planifolia* and *Vanilla* × *tahitensis*: Taxonomy and Historical Notes, Reproductive Biology, and Metabolites

**DOI:** 10.3390/plants11233311

**Published:** 2022-11-30

**Authors:** Renatha Tavares de Oliveira, Joana Paula da Silva Oliveira, Andrea Furtado Macedo

**Affiliations:** Integrated Laboratory of Plant Biology (LIBV), Institute of Biosciences, Federal University of the State of Rio de Janeiro—UNIRIO, Avenida Pasteur, 458, Urca, Rio de Janeiro 22290-240, Brazil

**Keywords:** food, fruits, phytochemistry, Orchidaceae, flavor

## Abstract

Vanilla is a worldwide cherished condiment, and its volatile market is associated with the so-called “vanilla crisis”. Even though only two species (*Vanilla planifolia* and *V.* × *tahitensis*) are cultivated on a large scale for commercial purposes, the *Vanilla* genus is comprised of 140 species. The present review article discusses the facets of this crisis, and vanilla crop wild relatives (WRs) are showcased as alternatives to overcome them. Historical, taxonomic, and reproductive biology aspects of the group were covered. Emphasis was given to the metabolic characterization of the vanilla crop WRs, highlighting their main chemical classes and the potential flavor descriptors. Many of these species can produce important flavor compounds such as vanillin, vanillic acid, and acetovanillone, among others. Overall, this review compiles valuable information that can help unravel new chapters of the history of this treasured product by evidencing the biotechnological potential of vanilla crop WRs.

## 1. Introduction

**Figure 1:**
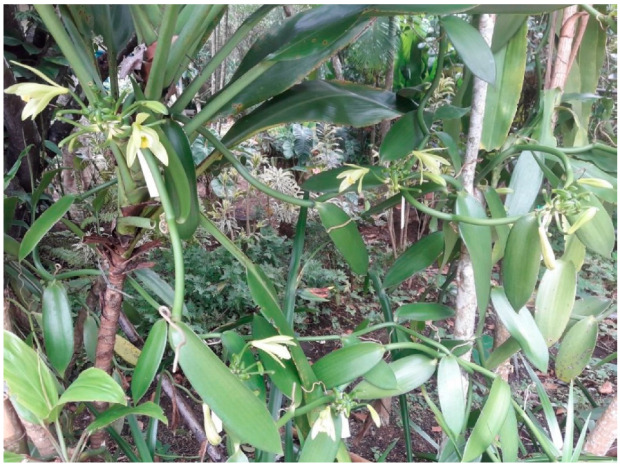
Habit of *Vanilla planifolia* in the Orchid Garden of the Botanical Garden of Rio de Janeiro, city of Rio de Janeiro (RJ, Brazil). Photo by: Aires Vanessa C. dos Santos.

The genus *Vanilla* Plumier ex. Mill (1754) (Orchidaceae) consists of 140 species, most of which are scientifically and commercially unexplored [1,2]. This genus, represented by perennial vines, is characterized by a thick and fleshy stem, a monopodial growth habit (Figure 1), aerial hairless roots growing at each node, and underground hairy roots, absence of pseudobulbs, alternate leaves, axillary inflorescence, flowers with lips partially adnate to the column, versatile anther that is generally saddle-shaped, and fruits with encrusted seeds [3,4]. Certain species of this genus are commercially designated as natural vanilla, a spice made from the fruit of orchid vines, which has a high gastronomic standard. Namely, they are *Vanilla. planifolia* Jacks. ex Andrews and *V.* × *tahithensis* J.W. Moore are among the market’s most expensive condiments [5,6].

**Figure 2:**
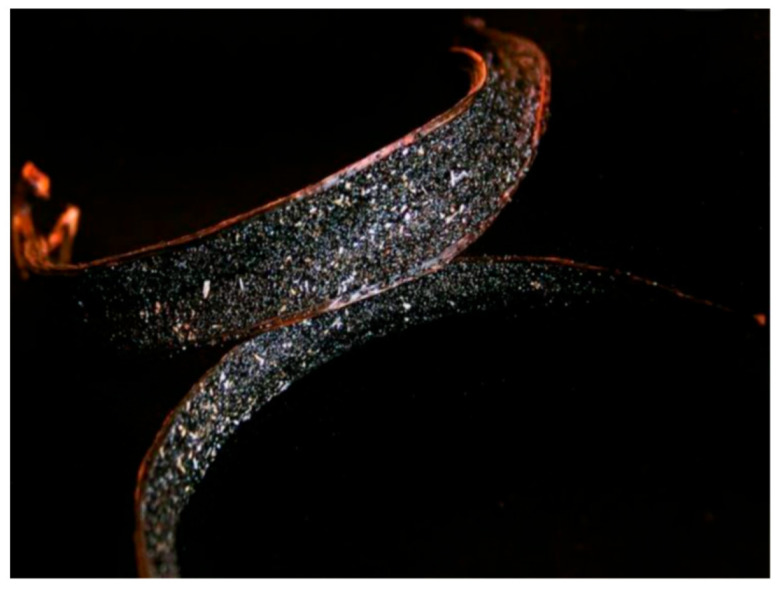
Open fruit of *Vanilla* sp. with vanillin crystals. Photo by: Marcelo Kuhlmann retrieved from “Frutos Atrativos do Cerrado” Project (http://www.frutosatrativosdocerrado.bio.br and @marcelo_kuhlmann Instagram profile, Accessed on 20 October 2022).

Vanilla, as a product, comprises a huge variety of sensorial experiences available for consumption. It can be purchased commercially from low-quality synthetic vanilla extracts (mainly artificial vanillin) to high-quality cured Madagascar vanilla fruits (capsules, beans, or pods). In exceptional-quality vanilla pods, vanillin crystals can be observed in the form of small white needles (called, in French, *givre*) (Figure 2). Despite this diversity of products available, with a very distinct sensory spectrum, it is important to highlight that the premium gastronomic quality of vanilla is linked to its natural source [7]. Natural vanilla has a characteristic flavor due to a complex mixture of more than 250 compounds [8,9]. Composing this flavor, some molecules in high concentrations are characteristic of *V. planifolia*, such as vanillin, vanillic acid, *p*-hydroxybenzaldehyde, and *p*-hydroxybenzoic acid. Meanwhile, anisyl alcohol, anisic acid, *p*-hydroxybenzyl, and protocatechuyl are characteristic of *V.* × *tahitensis* [10,11]. Although both commercial species have qualitative similarities in their metabolic composition, for example, producing vanillin, quantitatively, they differ significantly. Vanillin makes up 80% of the total aromatics in *V. planifolia* and 50% of the total aromatics in *V.* × *tahitensis* [10]. Despite the quantitative differences between commercial species, the concentration of flavor molecules is not proportionally linked to their contribution to the final sensorial perception of flavor and aroma [9]. This applies to many molecules, namely guaiacol, 4-methylguaiacol, acetovanilone, and vanillic alcohol found in small amounts in *V. planifolia*, but demonstrably perceived as intensely as vanillin [11].

Despite the high added value of this spice, which is crucial in several industrial segments, including culinary, cosmetic, and medicinal industries, its market faces serious supply problems [12,13]. In 2018, the world experienced what has been called the “vanilla crisis”, which promoted an unbridled increase in the price of natural vanilla in the global market, reaching values above silver [14]. This “crisis” was a consequence of a steady increase in demand, with a concomitant reduction in global supply. The causes for the natural vanilla global supply decline are cultivation-related. Vanilla cultivation is heavily based on the clonal material from a single species (*V. planifolia*). This species’ gene pool undergoes one of the most impressive processes of genetic erosion, being limited by, and susceptible to, biotic and abiotic stresses. In addition, the center of species diversity in Mexico is under intense anthropogenic pressure, and renewal of planted varieties is increasingly unlikely [6]. Many of vanilla’s suitable and/or native biomes are threatened and changes in tree species composition can bring irreparable damage to vanilla communities, as these semi-terrestrial orchids are found on shrubs and trees [15]. Furthermore, the agricultural production of vanilla occurs predominantly in only a few countries outside the Neotropics, the native growing area of aromatic vanilla [12]. Global demand for vanillin, the main molecule in vanilla flavor, is expected to grow by 6.2% by 2025, and the global vanillin market is expected to reach $724.5 million in the same year [16]. Meanwhile, up to 75% of the world’s natural vanilla comes from small farms on the island of Madagascar [1]. 

Although the historically reduced supply of natural vanilla has suffered significant losses, consumers’ search for healthier products with natural ingredients has led large companies (Hershey’s, Nestlé, Kellogg’s, General Mills) to seek natural sources of this flavor [17]. In 2018, the U.S. was the world’s top importer, according to the Observatory of Economic Complexity [18]. Nevertheless, *V. planifolia*, the main natural source of vanillin, can supply less than 1% of the annual market demand [19]. Other species of the genus, *V. × tahitensis* and *V. pompona* Schiede, are also produced for commercial purposes, although with minor distribution [7]. Disturbingly, many products labeled as “vanilla” are not made exclusively with vanilla beans but are complemented with synthetic molecules or trace amounts of natural vanilla. The number of lawsuits issued by consumers against companies that falsely advertise their products as “vanilla” has grown enormously in the US [18]. 

Scientifically understanding the chemical diversity of this genus and its floral biology is essential for the development of strategies aimed at increasing the natural production of vanilla. Despite the economic importance of this crop, relatively little attention has been given to its wild relatives (WRs), particularly concerning their biology and potential use [12]. Crop WRs are recognized as important resources for maintaining global food security and promoting sustainable rural livelihoods in the face of climate change [20]. Commercially unexplored *Vanilla* wild species can be sustainably introduced directly to the market, with the benefit of the presence of natural pollinators in their native environment [21]. Also, Vanilla crop WRs may be a source of new and useful phenotypic traits for the improvement of commercial species, such as adaptation to climate change, disease resistance, and product quality and stability. The establishment of vanilla crop WRs in the market most likely depends on farmers gaining experience in growing and curing high-quality fruits, a centuries-old knowledge that accompanies *V. planifolia* cultivation history [7]. This is already happening, precariously and occasionally, in some regions of Brazil, where the vanilla species grown in the central-west region of the country are recognized for their gastronomic value. However, like many orchids, most wild vanilla species are under pressure, especially due to deforestation and unsustainable collection [12].

Considering the scenario presented above, the objective of this review is to showcase the reproductive biology of some species of the genus, other than the established commercial species, and their chemical composition. Some aspects of taxonomic classification will also be addressed. Unknown and poorly classified accessions are common in *Vanilla* because species can be very similar to each other, and morphological characteristics can vary during development and between different environments. Flower morphology usually supports a species designation, but flowering can be an infrequent and erratic event [22].

## 2. Historical and Taxonomic Notes

*Vanilla* fruits have been used as a flavoring and medicinal beverage since the Preclassical period by multiple cultures in Mesoamerica, including the Mayans, Olmecs, Aztecs, and Totonacs (civilizations of present-day Mexican territory), most notably in ‘atole’, a corn-based drink. Vanilla was considered sacred by the native peoples of these regions and used to perfume their temples. The Mayans also created a drink derived from cocoa and it was probably at this time that vanilla began to be used as a spice to flavor food [1,23]. Documents have shown that *V. planifolia* Jacks. ex Andrews was chosen from over 100 species and first domesticated by the Aztecs in the Postclassical period because of its flavoring properties. Until recently, it was believed that vanilla was only used in these regions, however, important chemical compounds from natural vanilla were found in ceramic vessels placed in a tomb dated to the Middle Bronze III in Israel. This was the first archaeological evidence of vanilla exploration in the ancient Old World, circa 1650–1550 BC [24]. In the 16th century, after the Spanish conquest of the Aztecs, vanilla was introduced in Europe but was not cultivated outside of its native range until 1832, when Edmond Albius, from Reunion Island, developed a technique for manually pollinating the flowers [13]. Genetic data confirmed that the origin of the vanilla cultivated worldwide was in Mexico, most precisely in the Papantla region [13]. Even though it was considered a flavor valued as a luxury product, vanilla came to have a real commercial value only in the 17th century, as a component of chocolate, which was popular in European capitals [7].

Currently, most *Vanilla* species (61 species) are in the Neotropical region, including South and Central America, Caribbean islands, and Southern Florida. *Vanilla* includes orchids with a hemiepiphytic growth habit. Many species develop in tropical forests; only a few are adapted to more arid conditions and only one leafless species is adapted to extreme drought [3]. Even though wind dispersal is common for the remarkably small seeds present in orchids’ dry capsular fruits, *Vanilla* seeds are associated with a moist pulp and depend on dispersion by animals, such as bees. Thus, with shorter dispersal distances than in wind-dispersed orchid species, the genetic drift in *Vanilla* is presumably more intense [25].

*Vanilla* and related orchids (15 genera) are classified within their unique subfamily, Vanilloideae, a monophyletic subfamily, belonging to the tribe Vanilleae and subtribe Vanillinae according to molecular phylogenetic studies. Vanilloideae is characterized by having flowers with a single fertile anther, such as Epidendroideae and Orchidoideae, the largest and second largest subfamily of Orchidaceae, respectively. However, this condition evolved independently of Epidendroideae and Orchidoideae and is the result of a unique mode of floral development [26].

*Vanilla* is divided into two subgenera, *Vanilla* and *Xanata*. Species with membranaceous leaves, inflorescences poorly distinguished from the vegetative axis, lack of penicillar callus on the labellum, a column united to the labellum only at the base, a concave stigma, and a sub-perpendicular anther comprise the *Vanilla* subgenus [4]. *Xanata* have leafless, coriaceous to fleshy leaves species, and are divided into two sections. The *Tethya* section consists of leafy and leafless African and Asian species, and Caribbean leafless species, and has fruits devoid of aroma, while the *Xanata* section is divided into six groups, and consists of American species, about twenty of which have aromatic fruits, particularly within the morphological groups *V. planifolia* and *V. pompona* [4]. 

*Vanilla* species have a wide global distribution. However, locally, *Vanilla* population is small [20]. Consequently, the representation of these individuals in biological collections is also rare [20]. Therefore, *Vanilla* poses a taxonomical challenge regarding different vegetative parts present in the same species [2]. Vanilla crop WRs from Section *Xanata* urgently need to be evaluated for their biotechnological potential and are a global priority for conservation actions. However, only nine species have been assessed for the IUCN Red List of Threatened Species [27]. Of these, *V*. *cribbiana* is considered critically endangered, seven are classified as endangered, and one is Data Deficient [20].

## 3. Reproductive Biology

The process of sexual reproduction in plants begins with a pollination event. Thus, knowledge about pollination requirements, breeding systems, and reproductive strategies is paramount to better understanding the functioning of the reproductive process [28]. Vanilla production depends on the workforce to carry out hand pollination of thousands of flowers, and therefore pollination studies and identification of natural pollinators are of potential financial relevance [29,30]. Furthermore, through pollination ecology studies and the investigation of the reproductive biology of wild *Vanilla* species, it is possible to provide guidance to its conservation and to develop methods that increase fruit production [31]. Nevertheless, studies in this field are still scarce and limited to some species [32]. 

Species may exhibit different mating systems. To assess the breeding system of *Vanilla* species, different tests have been applied, such as (i) agamospermy, in which the pollen content was removed before the complete development of the flowers, as performed with *V. bicolor* flowers [33]; (ii) spontaneous self-pollination or autogamy experimentation, in which pre-anthesis buds are bagged for avoidance of a pollinator visit, using insect-proof bags; (iii) open pollination or control treatment, in which flowers remain under natural conditions and natural fruit set can be observed; (iv) manual self-pollination, in which flowers are pollinated with their own pollen, in order to investigate self-compatibility; (v) manual geitonogamy, which involves the pollen of a flower fertilizing another flower from the same parent plant or from a clone; (vi) manual cross-pollination, in which flowers are previously emasculated and then manually pollinated with the pollen from another individual; and (vii) natural cross-pollination, with emasculated flowers left under natural conditions [34,35,36,37,38,39,40,41,42]. Among these treatments, manual self-pollination and manual cross-pollination are usually more successful in fruit production and exhibit high fruit sets. Meanwhile, with hand self-pollination, fruit set can reach up to 100% in *V. paulista* [36], *V. barbellata*, *V. claviculata*, *V. dilloniana*, and *V. poitaei* (Panetto & J.D. Ackerman, unpubl. data and L. R. Nielsen & J. D. Ackerman, unpubl. data) [43]. 

### 3.1. Vanilla Flowers

**Figure 3:**
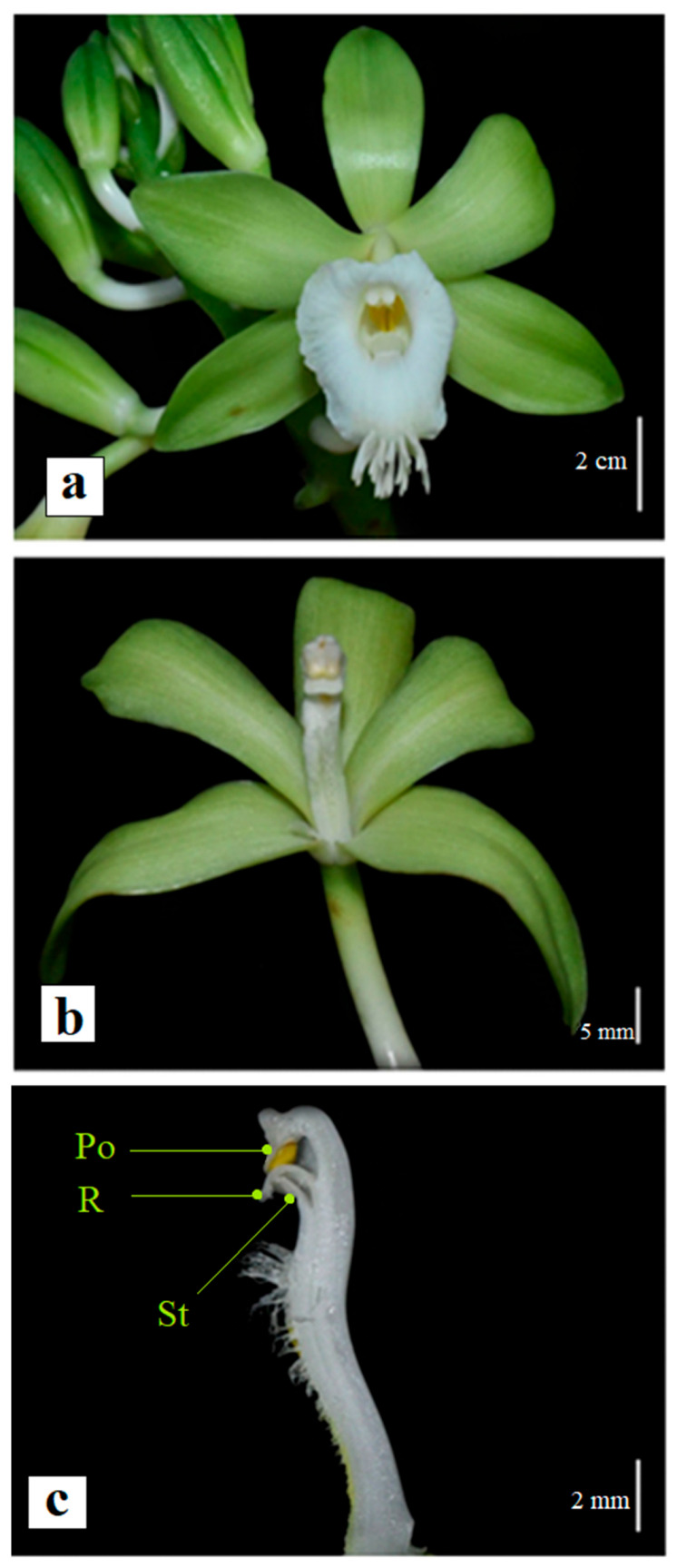
(**a**) Flower and floral buds of *Vanilla siamensis*. (**b**) Flower after labellum removal, with the column in evidence. (**c**) Longitudinal section of the column showing the anther bearing the pollen mass (Po), rostellum membrane (R), and stigma (St). The figure was adapted from Chaipanich et al. [51].

*Vanilla* flowers are predominantly large and present colors varying from green, white, yellow, and purple or a combination of these tones [44]. Axillary inflorescences produce resupinate flowers, in which the downward labellum is generally united to the column forming a floral tube and a landing platform for pollinators (Figure 3a) [4,44]. The flowers exhibit an anther bending downward (Figure 3b) [44,45], in which the pollen content is separated from the stigma by the rostellum (Figure 3c), a membrane that forms a physical barrier between the female and male reproductive systems, avoiding self-pollination [44,46,47]. The rostellum can be absent, as in *V. inodora* and *V. guianensis* flowers [48,49]. The labellum of *Vanilla* blossoms may have fleshy hairs, papillae, trichomes, penicillate callus, or a callus formed by a longitudinal keel or fleshy cushions [48,50]. The South American species of *V. planifolia* group (Subgenus *Xanata*, Section *Xanata*) have a penicillate callus at the middle portion of the labellum, positioned just below the anther and stigma [32,50]. As discussed in Pansarin’s study [32], although the presence of these structures is commonly associated with pollinator attraction, the callus may have a role in the pollination process by lifting the body of the pollinator and thus facilitating its contact with the anther, as reported for *V. paulista*.

Inflorescences are usually raceme, lateral, axillary, or terminal [2,52]. The number of flowers per inflorescence varies among species, which can be multiflowered, few-flowered, or carry an average amount of flowers. For instance, the inflorescences of *V. hostmanii* from Amazonia, *V. bahiana,* and *V. planifolia* bear up to 50, 31, and 26 flowers, respectively [40,48], while *V. edwallii* and *V. methonica* inflorescences bear, respectively, 1 to 4 flowers [53] and 4 to 5 flowers [52]. Generally, a single flower opens per inflorescence per day, as reported for *V. planifolia*, though this number also varies between species. Some leafless species from Madagascar, *V. bosseri*, *V. decaryana,* and *V. madagascariensis* present up to three open flowers per inflorescence per day [2,4]. *Vanilla* flowers are ephemeral and usually last a single day, as in the case of the species *V. odorata*, *V. insignis*, *V. helleri*, *V. hartii*, *V. planifolia* [48], *V. palmarum* [49], *V. paulista* [36], *V. bahiana*, *V. bicolor*, *V. phaeantha*, and *V. ribeiroi* [32]. For instance, *V. odorata* flowers remain open from 7 to 16 h, until 2:30 P.M., and *V. insignis* flowers from 8 to 14 h [48]. However, some species have long-lived flowers, such as *V. inodora*, the flowers of which last 2–3 days [48]; *V. imperialis*, the flowers of which remain in good conditions for reproduction for 4–5 days [46]; *V. guianensis* flowers remain open for 3–7 days [49]; *V. siamensis* flowers last up to 3 days [51]; *V. chamissonis* and *V. pompona* flowers last about 2 days; and *V. edwallii* flowers last up to 7 days [32]. 

### 3.2. Breeding System and Pollination

The pollination of *Vanilla* flowers occurs with pollen transference from the anther to the stigmatic cavity of the same or distinct flower [44]. Most *Vanilla* species, such as *V. planifolia*, are self-compatible but depend on a pollinator to promote sexual reproduction, due to the floral morphology that prevents spontaneous self-pollination [54]. Natural pollination events rarely occur; thus, natural fruit set is low, except for a few autogamous species which will be further discussed [47]. Low natural fruit set (around 1%) has been reported for *V. planifolia*, even in Mexico, where vanilla is native and natural pollinators are present [55,56,57]. Therefore, hand pollination is required for fruit production and, since its emergence in the XIX century (see Section 2), was adopted worldwide and remains essential in vanilla culture [55,58]. Low rates of fructification under natural conditions were also observed in other species: *V. humblotii* (0.62–1.2%) [39]; *V. ribeiroi* (1.1%) [49]; *V. bahiana* (2.35%) [40]; *V. pompona* (2.42% and 5%) [41,57]; *V. bosseri* (3.96%) [37]; *V. cristato-callosa* (6.6%) [49]; *V. poitaei* (6.4%); *V. dilloniana* (14.5%); *V. claviculata* (15%); *V. barbellata* (18.2%) [43]; and *V. edwallii* (<15%) [53]. A wild leafless species from South Africa, *V. roscheri*, despite pollinator dependence and the presence of a large rostellum covering the entire stigmatic surface, presents the largest natural fruit set (26.3%) ever recorded for a non-spontaneous self-pollinating *Vanilla* species [35]. Gigant and co-workers suggested that the higher fruit set observed in *V. roscheri* was due to the high abundance of visitors and the effectiveness of the potential pollinators, the visits of which involved pollen movements [35].

According to Soto Arenas [56], lower rates of flower visitants and low fruit set of *V. planifolia* in natural conditions are related to the food deception mechanism, which was also reported for *V. insignis* and *V. odorata*. The deceptive mechanism is widely accepted for *Vanilla* species and was also suggested for *V. paulista* [36], *V. bahiana* [40], *V. bosseri* [37], *V. edwallii* [53], *V. humblotii* [39], *V. siamensis* [51], *V. pompona* [41], and *V. grandiflora* [29]. Pollination by food deception is common to many orchid flowers, in which they appear to offer the pollinators food, but are rewardless [59]. Recent research clarified the reproductive strategies of some species with interesting findings. Pansarin [32] demonstrated that species previously thought to be rewardless and pollinated through food deception, such as *V. bahiana* and *V. pompona*, produce nectar in a nectar chamber in the labellum. Nectar secretion was also observed in *V. bicolor*, *V. chamissonis*, *V. hartii,* and *V. phaeantha* flowers, which offer nectar as a floral reward [32]. In another recent study, *V. palmarum* flowers exhibited nectar production and storage, and secretory cells longitudinally disposed in the labellum. This species’ flowers seem to be adapted to hummingbirds’ pollination [42]. According to Pansarin [32], the new findings regarding the pollinator attraction mechanism of *Vanilla* flowers suggest that the previous assumption of a reward absence is possibly due to a scarcity of information and the limited number of studied species.

Unlike outcrossing species, a high natural fruit set is commonly reported for autogamous species [29,46]. The following *Vanilla* species are thought to be spontaneous self-pollinators due to their high fruit set under natural conditions: *V. martinezii* (up to 53% in a clone) [48], *V. guianensis* (78%) [49], *V. Mexicana* (syn. *V. inodora*) (53.9%) [38], *V. bicolor* (42.5 and 71%) [33,49], *V. savannarum*, *V. griffithii* [50], and *V. palmarum* (70.3%) [49,50]. Different mechanisms were proposed by researchers to explain spontaneous self-pollination in *Vanilla* flowers: (i) the leaking of an abundant stigmatic fluid, which promotes its contact with the pollen grains, and induces the germination of pollen tubes [33,60]; (ii) the presence of a reduced or dehydrated rostellum that facilitates the encounter of pollen and stigma [33]; and (iii) the contact of the growing anther and stigma in early anthesis, as observed in *V. guianensis* [49]. 

In the Peruvian Amazon, where *V. bicolor* exhibits cleistogamous flowers (flowers that remain closed), it was reported that a high natural fruit set (42.5%) and a high fruit set (71%) at spontaneous auto-pollination treatment (bagged flowers) were associated with excessive stigmatic fluids and a thin rostellum [33,49]. Pansarin [32] reported that Brazilian individuals of *V. bicolor* are chasmogamous (with open flowers) and observed self-fertilization assisted by rain. Gigant et al. [38] observed a high natural fruit set (53.7%) of *V. mexicana* and a similar fruit set with bagged flowers (53.9%), which indicates that the species do not depend on a pollinator. Analysis of *V. mexicana* autogamous flowers verified the presence of pollen adhered to a glandulous and sticky rostellum, which might indicate a stigmatic leak [38]. High pollination rates were reported for *V. palmarum* (70.3%) in Peru and self-pollination was also associated with the leaking of excessive stigmatic fluids [49]. In Brazil, recent records of *V. palmarum* indicated that flowers produce nectar and are pollinated by hummingbirds, but autogamy may also occur. The fruit set of Brazilian populations of *V. palmarum* was high in both natural (67.3–71.4%) and bagging (66.6–73.3%) experiment conditions [42]. In *V. humblotii*, despite the presence of a large rostellum covering the stigma surface and the pollinator dependence, unexplained spontaneous self-fertilization was reported, with 6.7% of fruit set [39], but the natural fruit set is low (~1%). On the other hand, in *V. chamissonis*, low rates of fruit set (6.06%) were observed in bagging experiments, while higher fruit sets are observed under natural conditions (21.21%) and through hand pollination (75.75% and 78.78%) [34]. Rodolphe et al. [46] suggested that the uncommon natural fruit set of *V. chamissonis* is associated with the strong fragrance released by these flowers.

### 3.3. Pollinators and Visitors

Although information regarding pollination and biological interactions of *Vanilla* are scarce [61] and natural pollination is still poorly understood [41], there have been recent efforts for their elucidation [32,35,36,37,39,40,41,42,49,51,53]. According to Childers and Cibes [55], hummingbirds and small *Melipona* bees were thought to be the pollinators of *Vanilla* in Mexico. However, as discussed by Lubinsky et al. [29] and pointed out by Dressler [59], due to the small size of *Melipona* bees, they do not seem to be capable of performing the required steps of pollination. Dressler [59] proposed that large bees of the *Eulaema* genus (Apidae: Euglossini) are the pollinators of *Vanilla* flowers in the American tropics, which was further confirmed for some vanilla species [29,40,41,49]. Soto Arenas [56] described three pollination systems for Mexican vanilla species. According to the author, *V. inodora* is pollinated by carpenter bees (*Xylocopa*), while *V. pompona*, *V. hameri,* and *V. cribbiana* produce fragrances, such as limonene, attracting male Euglossini bees, and *V. insignis*, *V. odorata*, and *V. planifolia* exhibit a deceptive mechanism [56]. Although birds and different insects are commonly observed visiting *Vanilla* flowers, bees are often considered potential or effective pollinators.

Bees were pointed out as the pollinator group of *V. barbellata*, *V. claviculata*, *V. dilloniana,* and *V. poitaei* by Panetto and Ackerman, and Nielsen and Ackerman in unpublished data [43]. In Thailand, *Thrinchostoma* sp. bees are a potential pollinator of *V. siamensis* [51]. In the Peruvian Amazon, a male *Eulaema meriana* was observed removing pollen from *V. grandiflora* flowers without exhibiting the usual scent-collection behavior of hovering and transferring scents to the hind tibia [29]. In Peru, *V. pompona* subsp. *grandiflora* flowers were pollinated by two Euglossini bees: *Eulaema meriana* and *Euglossa imperialis* [49]. In Costa Rica and Peru, male bees of *Eulaema cingulata* are considered effective pollinators of *V. pompona* flowers, as pollen masses were observed in their scutellum after looking for nectar inside the labellum. In this case, a dual mechanism was suggested: a food deceptive mechanism due to nectar absence, and the offering of fragrances as a reward, due to the scent-collection behavior of *Euglossa* and *Eulaema* bees on the tepals of the flowers [41]. The flowers of *V. pompona* are fragrant and release twenty floral volatile compounds, with trans-carvone oxide, limonene, and limonene oxide as the major ones, and the first one is associated with the attraction of *Eulaema* bees [41]. Soto Arenas and Dressler [48] reported a clover scent in *V. dressleri* flowers which was associated with pollination by male Euglossini bees. In Brazil, Pansarin and Pansarin [36] reported pollination of *V. paulista* flowers by males of *Eulaema nigrita* and *Eufrisea violacea*, which occurred when the bees left the flowers and the pollen mass attached to their bodies were deposited on the stigma aided by the rostellar flap. Also in Brazil, a brief visit of *Eulaema* sp. resulted in fruit formation and male *Epicharis affinis* (Apidae: Centridini) bees were observed carrying the pollen of *Vanilla.* Thus, they were considered effective pollinators of *V. bahiana* and *V. edwallii,* respectively [40,53]. In both cases, the authors suggested that pollination occurs through a deceptive mechanism. Although *V. edwallii* flowers release a sweet fragrance that attracts and keeps bees patrolling the flowers, the fragrance does not seem to be harvestable, as reported by Pansarin and collaborators [53]. Three different female bees were considered as potential pollinators of *V. roscheri* species in South Africa and were associated with its high natural fruit set: two allodapine bees, *Allodapula variegate*, *Allodape rufogastra* (Apidae: Xylocopinae, Allodapini)*,* and an anthophorine bee (Apidae: Apinae: Anthoporini) [35]. On the other hand, Petersson [37] proposed that the major visitor of *V. bosseri* in Madagascar, an allodapine bee, is a pollen thief that might occasionally pollinate the flowers. On Mayotte Island, only a few visits were observed in *V. humblotii* flowers by a female allodapine bee and a female sunbird (*Nectarinia coquerelli*). The sunbird was foraging insects and small invertebrates within the flowers [39]. 

**Figure 4:**
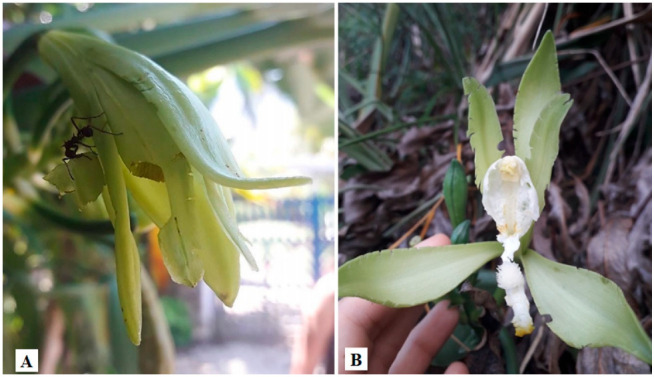
Ants’ predatory behavior in *Vanilla* flowers in Rio de Janeiro, Brazil. (**A**) Ant with predatory behavior in *Vanilla planifolia* flower; (**B**) *Vanilla bahiana* flower damaged by ants. Photos by: (**A**) Aíres Vanessa Cavalcante dos Santos; (**B**) Renatha Tavares de Oliveira.

Visits by birds were also observed in *V. planifolia* flowers, which were occasionally visited by hummingbirds [29], and visits by the bird *Zosterops* were observed in vanilla plantations in Reunion Island [62]. In a recent study, Pansarin and Ferreira [42] observed a species of hummingbird (*Amazilia fimbriata*) pollinating *V. palmarum* flowers. The birds were observed hovering in front of the inflorescences, landing on the labellum apex, and placing their head inside the floral tube, which contained nectar in the nectar chamber. Despite the presence of an effective pollinator, *V. palmarum* is self-compatible and shows high fruit set in spontaneous self-pollination treatment (66.6–73.3%), revealing that is not pollinator-dependent. Different insect groups, such as Blattodea, Coleoptera, Diptera, Hemiptera, Hymenoptera, Lepidoptera, Mantodea, and Orthoptera, have been observed visiting *Vanilla* flowers [40,51]. Ants are commonly reported as frequent visitors of vanilla flowers, but they are not assumed as potential pollinators [29,34,39,40,49,51]. The ants are attracted by the extrafloral nectar produced during bud development, at the abscission layer between the bud and ovary [46,49]. Householder et al. [49] observed ants feeding on these sugary exudates of *V. cristato-callosa* in Peru. In Brazil, Anjos et al. [40] observed ants protecting the flowers of *V. bahiana* and chasing away insects of Diptera, Coleoptera, and Orthoptera. On the other hand, our study group observed floral herbivory by ants in flowers of *V. planifolia* (Figure 4A) and *V. bahiana* (Figure 4B), both in Rio de Janeiro, Brazil. Predatory behavior of ants (*Acromyrmex octospinosus*) was also observed in *V. mexicana* flowers in Guadeloupe, causing great damage [38]. Moreover, floral herbivory by Coleoptera and Orthoptera insects was observed in *V. bahiana* flowers [40]. Altogether, the compilation of data on vanilla crop WRs fruit sets respective of breeding systems, pollination strategies, and potential pollinators is hereby presented (Table 1).

## 4. Chemical Traits from Vanilla Crop WRs

All the comprehensive and up-to-date taxonomic and reproductive biology knowledge reported in the previous sections leads to the subject of the feasibility of the vanilla crop WRs as alternatives to overcome the so-called “vanilla crisis”. Despite the FDA’s “standard of identity” definition of vanilla beans destined for human consumption as the dried cured fruits of *Vanilla planifolia* Andrews and *Vanilla* × *tahitensis* Moore (US Code of Federal Regulations for Vanilla, 21 CFR 169.3), the study of their WRs can be advantageous in many realms [47,63,64]. It is understood that breeding strategies provide an impulse toward the enhancement of vanilla production which would otherwise be subject to crop loss [65]. Also, as the commercial species have very restricted germplasm, traits from WRs can be targeted for their improvement. Reportedly, *V.* × *tahitensis* benefitted from the natural hybridization process by incorporating important traits from its ancestor *V. odorata*, such as indehiscent pods by the time of maturation. The “Vaitsy” cultivar type, likely a hybrid of *V. planifolia* and *V. pompona,* is resistant to the *Fusarium* fungus, which, as mentioned, is a great threat to vanilla crops [66,67,68]. Yet not only are phenotypic hybridization features related to enhanced crop productivity or pathogen resistance, but molecular improvements are also desired. Reportedly, *V. planifolia* and *V.* × *tahitensis* Haapape hybrids produce a higher percentage of vanillin than the common commercial species, besides developing indehiscent mature beans [68]. Also, the hybrid of *V. planifolia* × *V. tahitensis* called “Manitra ampotony”, cultivated in Madagascar, produces approximately 2.7 times more vanillin than common vanilla [68]. Hybrids of *V. planifolia* × *V. phaeantha* also showed a distinguished chemical profile compared to *V. planifolia*. Levels of vanillin and vanillyl alcohol in these hybrids were lower, though those of *p*-hydroxybenzoic alcohol were higher than in *V. planifolia* and the presence of anisyl alcohol, as in *V. bahiana*, was also reported [6]. 

In this section, metabolites reported in the surveyed bibliography were organized concerning the non-commercial and/or vanilla crop WR species in which they were found, the acquisition method applied, and their PubChem CID identifier. With the latter, it was possible to associate each molecule with its respective InChI and/or SMILES identifier, which could be correlated with chemical classes through the Classyfire tool [69]. This strategy enabled an overarching comparison of all compiled metabolite data. The PubChem CID identifier also favored the unification of molecule registries, since synonyms and CAS numbers were often absent or ambiguous within the surveyed studies. Finally, PubChem identifiers were also searched in the Flavor DB and associated with flavor descriptors if present [70]. The Flavor DB is a repository that assembles information on 25,595+ flavor molecules from several other databases, such as FooDB, Flavornet, and Fenaroli’s Handbook of Flavor Ingredients [70]. With this effort, we hoped to unify and enrich data on molecules found in vanilla species hereby surveyed that did not undergo any sort of olfactometry or volatile/flavor compound-focused study. We strongly encourage consulting data from those studies that did apply such techniques directly, as in the case of the hereby often-cited thesis by Galeas [71] that identified aroma molecules in *V. pompona* through gas chromatography-olfactometry (GC-O) analysis. The author [71] performed a greatly comprehensive study and herein their data are only briefly alluded to, as was the case of the extensive review of “Volatile Compounds in Vanilla” by Toth et al. [72] and others. Also, the assessed molecules were not limited to being found in fruits but also leaves and flowers (Table 2). Nonetheless, only molecules found in fruits underwent analysis with the FlavorDB.

### Molecules, Chemical Classes, and Potential Flavor Descriptors in Vanilla Wild-Relative Species

A total of 313 metabolites were compiled from the published literature pertaining to wild-relative and/or non-commercial vanilla species (Supplementary Table S1). Of these, 167 compounds were associated with flavor descriptors from the Flavor DB [70]. According to the Classyfire ontological classification, 33 chemical classes, 63 subclasses, and 129 direct parent classes were associated with the compiled molecules (Supplementary Table S1). Additionally, ten compounds could not be associated with unequivocal PubChem CIDs (5,7-dihydroxy-2-(3-hydroxy-4-methoxy-phenyl)-3-methoxy-chromone, 5-vinyl-guaiacol, anisyl palmitate, dracunculifoside J, glucoside A, glucoside B, hydroxydi-hydromaltol, methyl-2-(4-hydroyphenoxy) benzoate, *p*-hydroxybenzyl alcohol glucoside, and γ-aminobutiric acid), and therefore could not be unambiguously associated with flavor descriptors through the Flavor DB.

**Figure 5:**
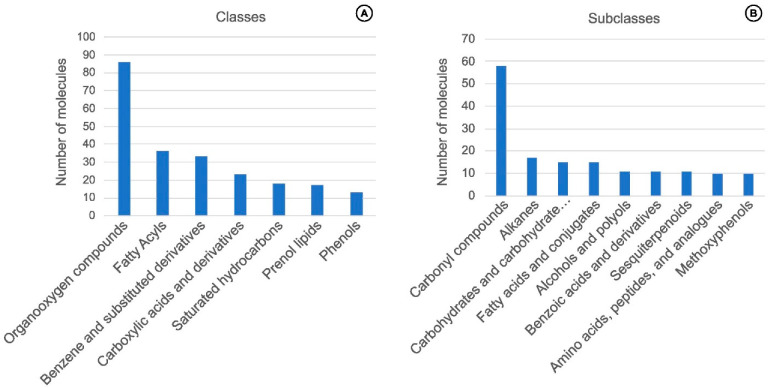
Pool of the most frequent chemical (**A**) classes and (**B**) subclasses correlated to compounds identified in vanilla crop WR and hybrid species.

Species with molecular information disclosed are *V. pompona*, *V. palmarum*, *V. ribeiroi*, *V. bahiana*, *V. chamissonis*, *V. sotoarenasii*, *V. crenulata*, *V. imperialis*, *V. planifolia* × *V. pompona* (hybrid), *V. planifolia* × *V. phaeantha* (hybrid), and a wild-type (likely *V. odorata* from Peru) [6,71,72,73,74,75,76,77]. From the hereby compiled data, the chemical classes of organooxygen compounds, fatty acyls, benzene and substituted derivatives, carboxylic acids and derivatives, saturated hydrocarbons, prenol lipids, and phenols had more than ten representative compounds (Figure 5A). The remaining 25 classes had seven or fewer associated compounds. The most frequent subclasses were carbonyl compounds, alkanes, carbohydrates and carbohydrate conjugates, fatty acids and conjugates, alcohols and polyols, benzoic acids and derivatives, sesquiterpenoids, amino acids, peptides and analogs, and methoxyphenols (Figure 5B). At last, the direct parent classes with the most representatives were alkanes, medium-chain aldehydes, methoxyphenols, and sesquiterpenoids (Supplementary Table S1). Such an overwhelming number of chemical features related to commercially poorly explored species are only a hint regarding their true biotechnological potential.

**Figure 6:**
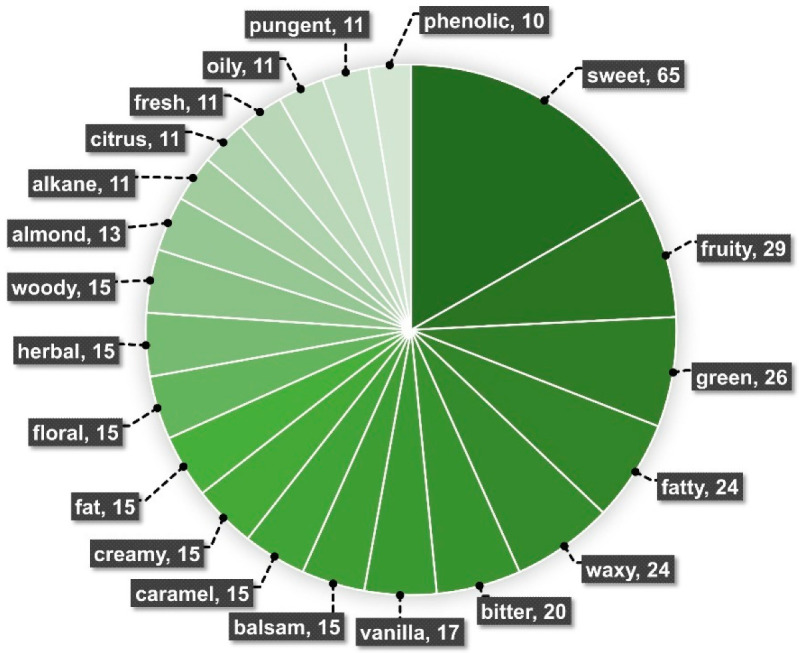
Pool of the most frequent flavor descriptors, retrieved from the Flavor DB, associated with molecules identified in the fruits of vanilla crop WR and hybrid species. The numbers represent the count in which each descriptor is reported.

Strikingly, 248 flavor descriptors were associated with molecules from WR vanilla species hereby surveyed through Flavor DB (Figure 6 and Supplementary Table S1). The ten most frequent flavor descriptors were sweet, fruity, green, fatty, waxy, bitter, vanilla, balsam, caramel, and creamy (Figure 6). By far, *V. pompona* is the wild-relative vanilla species with most studies related to the elucidation of its metabolites [6,71,72,75,76]. Both *V. pompona* subsp *grandiflora* (leaves and green pods) and *V. pompona* subsp *pittieri* (green pods), as well as cured *V. pompona*, were targets of chemical profiling studies (Supplementary Table S1). Molecules associated with the cherished commercial vanilla flavor are found in *V. pompona*, such as *p*-hydroxybenzaldehyde, *p*-hydroxybenzoic acid, *p*-hydroxybenzyl alcohol, *p*-anisyl alcohol, vanillic acid, vanillin, and vanillyl alcohol (Table 3 and Supplementary Table S1). From *V. pompona* cured pods, Galeas [71] associated through GC-O analysis the following compounds with the respective most impactful aroma descriptors: 1,5Z-octadien-3-one (geranium, pungent, plastic, vial, terpenic, green, fatty, fruity, familiar, candy, sweet, powdery, floral, strong); acetic acid (sour, sulfury, acidic, fatty, sweet, brown); trans-methyl cinnamate (sweet, phenolic, spicy, benzaldehyde, anisic, powdery, cherry, woody); 2-acetyl-1-pyrroline (popcorn, hazelnut, pretzel, cooked, baked, roast); 1-octen-3-one 3 (mushroom, earthy, vegetable, strong, green, herbaceous); 2-methyl-3-furanthiol (alliaceous, sulfury, bready, baked, pyrazinic, cracker like, earthy, savory, meaty); ethyl pyrazine (weird, sweet, veggie, plastic, phenolic, fruity, berry, redfruit, cherry, solventy, earthy); hexanal (green, fresh, grassy, ethereal); 3-methyl butyric acid (butyric [long], butyric, rancid, skunky, sour, cheesy, acidic); and 3Z-nonenal (citrusy, aldehyde, sweet, fruity, green, fatty, mix of fruits, pyrazinic, bread crust, oily, waxy oily, solventy). Even though vanillin was annotated with high signal intensity from GC-MS data acquired prior to GC-O, it was not among the molecules with the most impactful aroma in cured *V. pompona* olfactometry analysis (with only 50% of the intensity of the most intense compound, 1,5Z-octadien-3-one) [71]. Vanillin was also the most intense flavor-related compound, identified through GC-MS, in *V. pompona* by Ehlers and Pfister [73], followed by *p*-anisyl alcohol and vanillic acid. Through high-performance liquid chromatography with diode array detection (HPLC-DAD), Maruenda et al. [75] reported glucovanillin as the most concentrated compound in Peruvian *V. pompona* ssp. *grandifolia* fruits, followed by anisyl alcohol, vanillin, *p*-hydroxybenzaldehyde, vanillyl alcohol, vanillic acid, *p*-hydroxybenzaldehyde, and *p*-hydroxybenzoic acid. It is also reported that the vanillin content in *V. pompona* ssp *grandiflora* can greatly vary depending on its geographical origin [6]. Accessions from Guadalupe present significantly higher vanillin content (>2 g/100 g·dm) than those from French Guiana and Central America (<0.11 g/100 g·dm) [6]. Furthermore, a comprehensive metabolite compilation was performed by Toth and collaborators [72], whereby data on *V. pompona* (from unknown origin and Madagascar) and a wild-type species from Peru were hereby included (Supplementary Table S1). As mentioned by the authors, the wild species morphologically resembled *V. odorata*, but without confirmation. From the unknown wild-type species, 86 compounds were annotated; 71 were annotated from *V. pompona* from Madagascar, and 8 from *V. pompona* of unknown origin (Supplementary Table S1 and Toth et al. [72]). Clearly, combined data show that *V. pompona*’s rich flavor diversity potential is certainly valuable from a biotechnological perspective.

The differential analysis applied to LC-MS/MS metabolomics data from Atlantic Forest vanillas (*V. bahiana* and *V. chamissonis*) also denotes an interesting chemical richness pattern [77]. Both species produce compounds with considerably higher intensities than the commercial species, *V. planifolia*. *V. bahiana* had higher signal intensity for acetovanillone, a compound also detected in *V. planifolia*, with a much lower intensity compared to vanillin. Nonetheless, by means of GC-O, it was observed that acetovanillone exerts an aroma intensity as strong as vanillin even with 1000 times less concentration in *V. planifolia* [11]. According to the Flavor DB, acetovanillone has the following flavor profile: “vanilla, sweet, vanillin, and faint”; while, as stated by Pérez-Silva et al. [11], its odor quality is “vanilla, sweet, and honey” when extracted from *V. planifolia* cured pods. In *V. chamissonis*, vanillic acid had higher signal intensity than in *V. planifolia* regarding negative ionization data from ultra-high performance liquid chromatography coupled with sequential mass spectrometry analysis (UHPLC-MS/MS) [77]. Vanillic acid composes one possible pathway for endogenous vanillin biosynthesis [78]. According to the Flavor DB, this molecule has the “powdery, vanilla, bean, milky, sweet, creamy, and dairy” flavor profiles associated with it. Moreover, other compounds from both species are associated with many potential flavor descriptors, which could impart a unique quality to them (Supplementary Table S1). Vanillin was annotated in both species, though in a much lesser concentration than *V. planifolia* [77]. In their research, Pérez-Silva and collaborators [6] quantified metabolites considered fundamental, for their aromatic potential, to compose the knowledge basis for future vanilla breeding programs. They annotated from *V. bahiana* the following vanilla flavor-related compounds: anisyl alcohol (highest content in g·100 g^−1^ dry weight), *p*-hydroxybenzoic alcohol (2nd highest), *p*-hydroxybenzoic acid (3rd highest), vanillic acid, vanillyl alcohol, and *p*-hydroxybenzaldehyde. From *V. crenulata*, the authors annotated vanillyl alcohol (highest content) and *p*-hydroxybenzoic acid; from *V. imperialis, p*-hydroxybenzoic acid (highest content), and *p*-hydroxybenzoic alcohol were annotated. All targeted flavor molecules were identified in the hybrids *V. planifolia* × *V. pompona* and *V. planifolia* × *V. phaeantha* (vanillin, vanillic acid, vanillyl alcohol, *p*-hydroxybenzaldehyde, *p*-hydroxybenzoic acid, *p*-hydroxybenzoic alcohol, and anisyl alcohol). From that, anisyl alcohol was the compound with the highest content in *V. planifolia* × *V. phaeantha*, followed by vanillin and *p*-hydroxybenzoic alcohol. While in *V. planifolia* × *V. pompona*, the content of vanillin was the highest, followed by anisyl alcohol and *p*-hydroxybenzoic alcohol, which had similar contents. *V. sotoarenasii*, in the same study, presented vanillyl alcohol as the most concentrated compound, followed by *p*-hydroxybenzoic acid and *p*-hydroxybenzoic alcohol [6]. From all targeted molecules, only vanillic acid was not annotated in *V. sotoarenasii.* The authors also investigated the presence of these flavor molecules in *V. lindmaniana*, nonetheless, none of them were annotated in the species [6]. Also, despite the presence of aromatic compounds, the authors refer to *V. crenulata* and *V. imperialis* as non-aromatic species, as was, more expectedly, the case for *V. lindmaniana* [6].

The volatile compounds associated with the fragrance of *V. pompona* flowers were also investigated [41]. Almost 80% of the total floral perfume was composed of *trans*-carvone, limonene, and limonene oxide, whereby the first was attributed to the successful attraction of *Eulaema* bee species by *V. pompona* [41]. Additionally, metabolomic studies of the leaves of vanilla WR species were also conducted [74,76]. Through a Nuclear Magnetic Resonance (NMR) analysis, it was observed that the metabolome of in vitro Cymbidium mosaic virus (CymMV) infected vanilla plants was differentially expressed compared to uninfected plants [74]. Leaves from the *V. pompona* accession infected with CymMV had higher levels of glucoside A, sucrose, glucose, and phenylpropanoid and flavonoid glucosides, compared to those of *V. planifolia*, *V. tahitensis*, and *V. planifolia* × *V. tahitensis*. These results combined with those of the growth performance of CymMV-infected vanilla plants led not only to the conclusion that *V. pompona* showed resistance against CymMV, but also that the infected species yield differential levels of target metabolites [74]. Hence, this study denotes the importance of the elucidation of metabolites to be used as biomarkers for the development of vanilla crops disease control protocols. The NMR-based metabolomic study by Leyva et al. [76] also aimed at the identification of molecular markers from vanilla leaves [76]. The authors assessed *V. planifolia*, *V. pompona*, *V. ribeiroi*, and *V. palmarum* differential metabolites expression through a multivariate statistics approach. They annotated 36 metabolites and found that the production of glucoside A significantly differs between vanilla-fragrant (*V. planifolia* and *V. pompona*) and vanilla-non-fragrant (*V. ribeiroi* and *V. palmarum*) species. The classes of organic acids, sugars, phenolic glucosides, and amino acids were considered the most relevant markers to differentiate the four species. *V. ribeiroi* showed significantly higher levels of amino acids (such as alanine, arginine, isoleucine, leucine, phenylalanine, and others); *V. palmarum* showed significantly higher levels of asparagine, essential in the metabolism of nitrogen transport and storage [76], and *V. pompona*, together with *V. planifolia*, was associated with the highest amounts of glucoside A [76]. Lastly, these studies indicate meaningful ecological use cases for the elucidation of vanilla WR species metabolites.

Altogether, despite the aforementioned “standard of identity”, *V. pompona*, *V. chamissonis*, and *V. bahiana* are already commercially cherished in Central and South America [63,79,80]. There is a bright future regarding the innumerable possibilities in vanilla production and market scope. Not only can breeding programs benefit from bleeding-edge knowledge about wild vanilla species, but these species themselves represent great opportunities for small and local producers and traditional communities [12]. Gradually, the entity “vanilla” unravels new chapters that now include the precious attributes of its biological diversity [79,80]. Moreover, the diversity of chemical classes hereby shown, even if unrelated to the vanilla flavor, represent a vast source of potential natural products. Vanillin and vanillic acid present in *V. planifolia* and other vanilla species are already interesting research targets for the pharmaceutical industry due to their therapeutic activities, such as antisickling pain relief, antianxiety and antidepressant qualities, protection against neurodegeneration, and lipid and blood glucose regulation [81]. Recent advances point toward breeding and hybridization efforts and local production of native species [6,12].

## Figures and Tables

**Table 1:** Fruit sets of vanilla crop WRs respective of breeding systems, different pollination treatments, and potential pollinators.

Species	Breeding System	Natural Fruit Set	Manual Self-Pollination Fruit Set	Manual Cross-Pollination Fruit Set	Spontaneous Self-Pollination Fruit Set	Potential Pollinators	References
*V. bahiana*	Outcrossing	2.35%	11.11–71.43%	24.44–83.33%	0	*Eulaema* sp. bees	[40]
*V. barbellata* Rchb. f.	Outcrossing	18.2%	100%	-	-	Bees	[43]
*V. bicolor*	Autogamy	43%	-	-	71%	-	[33,49]
*V. bosseri*	Outcrossing	3.96%	86%	43%	0	Bees (*Macrogalea ellioti*, *Liotrigona modecassa*, *Liotrigona mahafalya*)	[37]
*V. claviculata*	Outcrossing	15%	100%	-	-	Bees	[43]
*V. chamissonis*	Outcrossing	21.21%	78.78%	75.76%	6.06%	-	[34]
*V. cristato-callosa*	Outcrossing	6.6%	-	-	-	Euglossine (*Euglossa* sp.) bees	[49]
*V. dilloniana*	Outcrossing	14.5%	100%	-	-	Bees	[43]
*V. edwallii*	Outcrossing	<15%	-	-	0	*Epicharis affinis* bees	[53]
*V. guianensis*	Autogamy	78%	-	-	-	-	[49]
*V. humblotii*	Outcrossing	0.62–1.2%	90.9%	86.7%	6.7%	*Allodape obscuripennis* bees and sunbird (*Nectarinia coquerelli*)	[39]
*V. martinezii*	Autogamy	up to 53% in a clone	-	-	-	-	[48]
*V. mexicana* (syn. *V. inodora*)	Autogamy	2.5–53.7%	-	-	53.9%	Carpenter bees (*Xylocopa* sp.)	[38,48]
*V. palmarum*	Autogamy	67.3–71.4%	76.7–80.0%	80–83.3%	66.6–73.3%	Hummingbirds (*Amazilia fimbriata*)	[42,49]
*V. paulista*	Outcrossing	0.4–6.9%	100%	-	0	*Eulaema nigrita* and *Eufriesea violacea*	[36]
*V. poitaei*	Outcrossing	6.4%	100%	-	-	Bees	[43]
*V. pompona*	Outcrossing	2.42% and 5%	-	-	0	*Eulaema cingulata* bees	[41,57]
*V. riberoi*	Outcrossing	1.1%	-	-	-	Euglossine bees	[49]
*V. roscheri*	Outcrossing	26.3%	64%	71%	0	Allodapine bees (*Allodapula variegata* and *Allodape rufogastra*)	[35]
*V. siamensis*	Outcrossing	3.6%	-	-	-	*Thrinchostoma* spp. bees	[51]

**Table 2:** Surveyed studies respective to hereby assessed vanilla crop WR or hybrid species and the plant organs from which the metabolites were extracted.

First Authors	Year of Publication	Species	Plant Organs
Ehlers and Pfister [73]	1997	*V. pompona*	fruits
Palama et al. [74]	2012	*V. pompona*	leaves
Maruenda et al. [75]	2013	*V. pompona*	fruits
Galeas [71]	2015	*V. pompona*	fruits
Toth et al. [72]	2018	*V. pompona* (from Madagascar)*, V. pompona* (origin unknown)*,* Wild Type (from Peru)	fruits
Leyva et al. [76]	2021	*V. pompona, V. palmarum, V. ribeiroi*	leaves
Pérez-Silva et al. [6]	2021	*V. pompona* subsp *grandiflora, V. pompona* subsp *pittieri, V. sotoarenasii, V. crenulata, V. imperialis, V. bahiana, V. lindmaniana, V. planifolia × V. pompona, V. planifolia × V. phaeantha*	fruits
da Silva Oliveira et al. [77]	2022	*V. bahiana, V. chamissonis*	fruits
Watteyn et al. [41]	2022	*V. pompona*	flowers

**Table 3:** Molecules commonly associated with the commercial natural vanilla flavor identified in the fruits of vanilla crop WR species. Numbers refer to respective references.

Species	*p*-Anisyl Alcohol	*p*-Hydroxybenzaldehyde	*p*-Hydroxybenzoic Acid	*p*-Hydroxybenzyl Alcohol	Vanillic Acid	Vanillin	Vanillyl Alcohol
*V. bahiana*	[6]	[6,77]	[6,77]	[6]	[6,77]	[77]	[6,77]
*V. chamissonis*		[77]	[77]		[77]	[77]	[77]
*V. crenulata*			[6]				[6]
*V. imperialis*			[6]	[6]			
*V. planifolia* × *V. phaeantha*	[6]	[6]	[6]	[6]	[6]	[6]	[6]
*V. planifolia* × *V. pompona*	[6]	[6]	[6]	[6]	[6]	[6]	[6]
*V. pompona* (from Madagascar)	[72]	[72]			[72]	[72]	
*V. pompona* (origin unknown)	[72]		[72]	[72]	[72]	[72]	[72]
*V. pompona* Shiede (cured)	[71,73]	[71,73]	[73]	[71]	[73]	[71,73]	
*V. pompona* subsp *grandiflora*	[6,75]	[6,75]	[6,75]	[6,75]	[6,75]	[6,75]	[6,75]
*V. pompona* subsp *pittieri*	[6]	[6]	[6]	[6]	[6]		[6]
*V. sotoarenasii*	[6]	[6]	[6]	[6]		[6]	[6]
Wild Type (from Peru)	[72]	[72]	[72]		[72]	[72]	[72]

## Data Availability

Not Applicable.

## Supplementary Materials

The following supporting information can be downloaded at: https://www.mdpi.com/article/10.3390/plants11233311/s1, Table S1: Metabolites identified from vanilla crop wild relatives and/or non-commercial vanilla species, following the alphabetical order of direct parent classes.

## Abbreviations

WRs—wild relatives; NMR—Nuclear Magnetic Resonance.
