# Supplementary material for: Vanilla beyond Vanilla planifolia and Vanilla × tahitensis: Taxonomy and Historical Notes, Reproductive Biology, and Metabolites
Source: Plants (Basel). 2022 Nov 30;11(23):3311. doi: 10.3390/plants11233311 (PMC9739750; doi:10.3390/plants11233311)
Supplement: Supplementary file 1 [file plants-11-03311-s001.zip › plants-2013316-supplementary.pdf]

Vanilla beyond *Vanilla planifolia* and *Vanilla* x *tahitensis*: taxonomy and historical notes, reproductive biology and metabolites

Renatha Tavares de Oliveira[1], Joana Paula da Silva Oliveira[1], Andrea Furtado Macedo[1]

1 - Integrated Laboratory of Plant Biology (LIBV), Institute of Biosciences, Federal University of the State of Rio de Janeiro

Supplementary Table S1. Metabolites identified from vanilla crop wild relatives and/or non-commercial vanilla species, following the alphabetical order of direct parent classes.

Metabolite	Species	Methods	Flavor DB descriptors	CID (PubChem)	Direct parent
2,3-butanediol	10c	GC-MS	onion, fruit, creamy, fruity, buttery	262	1,2-diols
phenol	8h, 15h, 10c	GC-MS, GC-O	phenol, plastic, rubber, phenolic	996	1-hydroxy-4-unsubstituted benzenoids
2,3-dihydroxypropyl acetate	8h	DTD-GC-MS		33510	1-monoacylglycerols
homoeriodictyol	1a, 2a	UHPLC-MS/MS	bitter	73635	3'-O-methylated flavonoids
viridiflorol	10c	GC-MS		11996452	5,10-cycloaromadendrane sesquiterpenoids
alpinumisoflavone	1a, 2a	UHPLC-MS/MS		5490139	6-prenylated isoflavanones
bletilol A	1a, 2a	UHPLC-MS/MS		102121477	7-O-methylated isoflavonoids
nevadensin	1a, 2a	UHPLC-MS/MS	if glucoside can be sweet-like	160921	8-O-methylated flavonoids
3-hydroxypropyl prop-2-enoate	15h	DTD-GC-MS		33101	Acrylic acid esters
methyl acrylate	8h, 10c, 15h	DTD-GC-MS, GC-MS		7294	Acrylic acid esters
cis-beta-ocimene	16i	GC-MS		5320250	Acyclic monoterpenoids
geranylacetone	10c	GC-O	magnolia, rose, fresh, floral, aldehydic, fruity, leaf, green	1549778	Acyclic monoterpenoids
linalool	16i	GC-MS		6549	Acyclic monoterpenoids
myrcene	16i	GC-MS		31253	Acyclic monoterpenoids

3-hydroxy-2-pentanone	10c	GC-MS	truffle, herbal, herb	62484	Acyloins
3-hydroxybutan-2-one	15h	DTD-GC-MS	butter, cream, milky, fatty, creamy, sweet, dairy, buttery	179	Acyloins
alanine	5d, 11d, 13d	NMR		5950	Alanine and derivatives
docosane	8h, 15h, 10c	GC-MS	alkane, waxy	12405	Alkanes
dodecane	10c	GC-MS	alkane	8182	Alkanes
eicosane	10c	GC-MS	alkane, waxy	8222	Alkanes
hentriacontane	15h	DTD-GC-MS		12410	Alkanes
heptacosane	8h, 15h, 10c	GC-MS		11636	Alkanes
heptadecane	10c	GC-MS	alkane	12398	Alkanes
heptane	10c	GC-MS	alkane, ethereal, sweet	8900	Alkanes
hexacosane	8h, 15h	DTD-GC-MS		12407	Alkanes
hexadecane	10c	GC-MS	alkane	11006	Alkanes
nonacosane	8h, 15h	DTD-GC-MS		12409	Alkanes
pentacosane	8h, 15h, 10c	GC-MS		12406	Alkanes
pentadecane	10c	GC-MS	alkane, waxy	12391	Alkanes
tetracosane	8h, 15h, 10c	GC-MS		12592	Alkanes
tetradecane	10c	GC-MS	alkane, waxy, mild	12389	Alkanes
tricosane	8h, 15h, 10c	GC-MS	alkane, waxy	12534	Alkanes
tridecane	10c	GC-MS	alkane	12388	Alkanes
undecane	10c	GC-MS	alkane	14257	Alkanes
2,4-dihydroxy-acetophenone	15h	DTD-GC-MS		6990	Alkyl-phenylketones

acetovanillone	1a, 2a, 15h, 10c	GC-MS, UHPLC-MS/MS, GC-O	vanilla, sweet, vanillin, faint	2214	Alkyl-phenylketones
o-acetyl-p-cresol	15h	DTD-GC-MS	floral, heavy, sweet, herbal	15068	Alkyl-phenylketones
propiophenone	10c	GC-MS	lilac, hawthorn	7148	Alkyl-phenylketones
threonine	5d, 11d, 13d	NMR		6288	Alpha amino acids
lactic acid	5d, 11d, 13d, 10c	GC-MS, NMR	acidic (if D or L)	612	Alpha hydroxy acids and derivatives
2,3-octanedione	10c	GC-MS	dill, buttery, broccoli, cooked	11449	Alpha-diketones
butane-2,3-dione	15h	DTD-GC-MS	butter, caramel, oily, creamy, sweet, pungent, strong	650	Alpha-diketones
hexane-2,3-dione	15h	DTD-GC-MS	butter, caramel, oily, fatty, sweet, creamy, fruity, phenolic, dry	19707	Alpha-diketones
pentane-2,3-dione	15h	DTD-GC-MS	butter, cream, caramel, sweet, pungent, creamy, nutty, cheese	11747	Alpha-diketones
3-methylpentanal	15h	DTD-GC-MS		27523	Alpha-hydrogen aldehydes
pyruvic alcohol	8h, 15h	DTD-GC-MS	caramellic, sweet, pungent, ethereal	8299	Alpha-hydroxy ketones
methyl pyruvate	10c	GC-MS		11748	Alpha-keto acids and derivatives
p-methylanisole	10c	GC-MS	powdery, cresol, naphthyl, camphoraceous, nutty, ylang, cedar	7731	Anisoles
arginine	1a, 2a, 5d, 11d, 13d	NMR, UHPLC-MS/MS	faint	6322	Arginine and derivatives
2-acetyl-5-methylthiophene	10c	GC-MS		83655	Aryl alkyl ketones
2-acetylpyrrole	10c	GC-MS, GC-O	nut, licorice, coumarin, bread, musty, nutty, walnut	14079	Aryl alkyl ketones

acetyl furan	8h	DTD-GC-MS	caramel, tobacco, balsamic, potato, coffee, sweet, cocoa, peanut, balsam, almond, beef	14505	Aryl alkyl ketones
2,5-furandicarboxaldehyde	10c	GC-MS		69980	Aryl-aldehydes
5-ethylfurfural	8h	DTD-GC-MS		89989	Aryl-aldehydes
5-hydroxymethylfurfural	1a, 2a, 8h, 10c, 15h	GC-MS, DTD-GC-MS, UHPLC-MS/MS	caramel, waxy, fatty, musty, cardboard	237332	Aryl-aldehydes
5-methylfurfural	8h, 15h, 10c	GC-MS	caramel, spice, burnt sugar, maple, almond	12097	Aryl-aldehydes
furfural	8h, 10c, 15h	DTD-GC-MS, GC-MS	fragrant, bread, woody, sweet, baked, almond	7362	Aryl-aldehydes
asparagine	5d, 11d, 13d	NMR		6267	Asparagine and derivatives
phenethanol	10c	GC-MS, GC-O	lilac, rose, rose water, honey, rose flower, floral, spice, bitter, rose dried	6054	Benzene and substituted derivatives
piperonal	9h, 10b	HPLC, DTD-GC-MS	coconut, heliotrope, powdery, sweet, vanilla, flower, violet, bitter	8438	Benzodioxoles
dihydroactinidiolide	10c	GC-MS	red fruit, coumarin, woody, musk, ripe apricot	27209	Benzofurans
dibutyl phthalate	10c	GC-MS	faint	3026	Benzoic acid esters
methyl benzoate	1a, 2a	UHPLC-MS/MS	prune, cananga, floral, wintergreen, lettuce, sweet, almond, herb, phenolic	7150	Benzoic acid esters
benzoic acid	10c	GC-MS	balsam, urine, faint	243	Benzoic acids
benzophenone	10c	GC-MS	peach, powdery, apricot, rose, balsam, geranium, metallic	3102	Benzophenones
benzaldehyde	15h, 10c	GC-MS	cherry, almond, sweet, burnt sugar, sharp, strong, bitter	240	Benzoyl derivatives

m-anisaldehyde	10b	HPLC	anisic	11569	Benzoyl derivatives
p-anisaldehyde	10bc, 15h	GC-MS, HPLC, HPLC-DAD, DTD-GC-MS, GC-O	mimosa, hawthorn, cherry, powdery, bitter, vanilla, anise, hawthorne, chocolate, balsam, creamy, minty, berry, sweet, floral, mint, almond, cinnamon	31244	Benzoyl derivatives
p-anisyl alcohol	1g, 6g, 7g, 8h, 9h, 10bc, 11eg, 12g, 14g, 15h	GC-MS, HPLC, HPLC-DAD, DTD-GC-MS, GC-O	caramel, chocolate, powdery, vanilla, rose, lilac, floral, sweet, honey, hawthorn, hyacinth, flower	7738	Benzyl alcohols
p-hydroxybenzyl alcohol	1g, 4g, 5d, 6g, 7g, 9h, 11deg, 12g, 13d, 14g, 10c	GC-MS, HPLC, HPLC-DAD, DTD-GC-MS	coconut, bitter, sweet, fruity, almond	125	Benzyl alcohols
benzaldehyde dimethyl acetal	10c	GC-MS	wine, sharp, floral, hummus, fruity, almond, green	62375	Benzylethers
(4-methoxyphenyl) methyl tetradecanoate	15h	DTD-GC-MS		18004148	Benzyloxycarbonyls
(4-methoxyphenyl)methyl hexadecanoate	8h, 15h	DTD-GC-MS		10809570	Benzyloxycarbonyls
(4-methoxyphenyl)methyl octadec-9-enoate	15h	DTD-GC-MS		10739892	Benzyloxycarbonyls
(4-methoxyphenyl)methyl octadecanoate	15h	DTD-GC-MS		23119703	Benzyloxycarbonyls
anisyl acetate	15h, 10c	GC-MS, GC-O	powdery, coumarin, vanilla, creamy, plum, sweet, balsam, fruity, cherry, almond, tonka	7695	Benzyloxycarbonyls
anisyl formate	15h, 10c	GC-MS, GC-O	vanilla, anisic, floral, fruity, seaweed	61054	Benzyloxycarbonyls
benzyl formate	10c	GC-MS	pineapple, apricot, floral, fruity, cranberry, almond, black tea, spicy	7708	Benzyloxycarbonyls

malic acid	5d, 11d, 13d	NMR		525	Beta hydroxy acids and derivatives
4-cyclopentene-1,3-dione	8h, 10c	GC-MS, DTD-GC-MS		70258	Beta-diketones
hentriacontene-2,4-dione	15h	DTD-GC-MS		56636502	Beta-diketones
heptacosene-2,4-dione	8h, 15h	DTD-GC-MS		53966535	Beta-diketones
nonacosene-2,4-dione	8h, 15h, 10c	GC-MS, DTD-GC-MS		16064344	Beta-diketones
nonadecane-2,4-dione	10c	GC-MS		567637	Beta-diketones
alpha-pinene oxide	16i	GC-MS		91508	Bicyclic monoterpenoids
I-R-alpha-pinene	16i	GC-MS		6654	Bicyclic monoterpenoids
(-)-beta-pinene	16i	GC-MS		440967	Bicyclic monoterpenoids
3,4-dimethyl-2,5-furandione	8h, 10c	GC-MS, DTD-GC-MS		13010	Butenolides
gibberellin A3	1a, 2a	UHPLC-MS/MS		6466	C19-gibberellin 6-carboxylic acids
butyl formate	10c	GC-MS	brandy, plum, fruity, rum	11614	Carboxylic acid esters
ethyl acetate	8h	DTD-GC-MS	pineapple, ethereal, sweet, anise, fruity, balsam, weedy, green	8857	Carboxylic acid esters
isopropyl acetate	8h	DTD-GC-MS	ethereal, chemical, banana, sweet, fruity	7915	Carboxylic acid esters
n-amyl formate	10c	GC-MS	ethereal, powerful, earthy, unripe banana, fruity, green	12529	Carboxylic acid esters
acetic acid	5d, 8h, 11d,13d, 15h, 10c	GC-MS, NMR	sour, pungent, sharp, vinegar	176	Carboxylic acids
formic acid	8h, 15h, 10c	GC-MS	pungent, vinegar, formyl	284	Carboxylic acids
isobutyric acid	10c	GC-MS	rancid, butter, strawberry, cheese	6590	Carboxylic acids

propionic acid	10c	GC-MS	cheesy, soy, acidic, vinegar, rancid, pungent	1032	Carboxylic acids
catechin	1a, 2a	UHPLC-MS/MS	bitter	9064	Catechins
cinchonain 1a	1a, 2a	UHPLC-MS/MS		442675	Catechins
catechol	8h, 10c	GC-MS		289	Catechols
7-hydroxy-2-methyl-4-oxo-4H-1-benzopyran-5-acetic acid	1a, 2a	UHPLC-MS/MS		14429402	Chromones
cinnamaldehyde	1a, 2a	UHPLC-MS/MS	spice, warm, red hots, paint, clove, sweet, candy, cinnamon	637511	Cinnamaldehydes
methyl-3-phenylprop-2-enoate	8h, 10c, 15h	DTD-GC-MS, GC-MS	balsam, sweet, cherry, strawberry	637520	Cinnamic acid esters
coumaran	8h	DTD-GC-MS		10329	Coumarans
sibiricose A1	1a, 2a	UHPLC-MS/MS		6326016	Coumaric acids and derivatives
coumarin	10b	HPLC	sweet, new mown hay, green, tonka, bitter	323	Coumarins and derivatives
2-ethylcyclobutanol	8h	DTD-GC-MS		37109	Cyclic alcohols and derivatives
2-hydroxy-2-cyclopenten-1-one	10c	GC-MS		82674	Cyclic ketones
cyclohexanone	8h, 15h	DTD-GC-MS	minty, acetone	7967	Cyclic ketones
1,3,5-trimethyl-cyclohexane	8h	DTD-GC-MS		35364	Cycloalkanes
bromocyclohexane	10c	GC-MS		7960	Cyclohexyl halides
methyl hexyl ether	8h	DTD-GC-MS	painty, herbal, sweet, fruity, lavender	78484	Dialkyl ethers
diisopropyl disulfide	10c	GC-O	cabbage, sulfurous, onion, alliaceous, meaty	77932	Dialkyldisulfides

fumaric acid	5d, 11d, 13d	NMR		444972	Dicarboxylic acids and derivatives
2,3-dihydro-2,5-dimethylfuran	8h	DTD-GC-MS		86510	Dihydrofurans
3H-pyran-2,6-dione	8h, 15h	DTD-GC-MS		574367	Dihydropyranones
5,6-dihydromaltol	10c	GC-MS		6429306	Dihydropyranones
5-hydroxy-5,6-dihydromaltol	10c	GC-MS		119838	Dihydropyranones
1,4-dimethoxybenzene	10c	GC-MS	sweet, fennel, bitter, green, new mown hay	9016	Dimethoxybenzenes
veratraldehyde	10c	GC-MS	caramel, vanilla, creamy, sweet, woody, cherry, minty	8419	Dimethoxybenzenes
veratryl alcohol	15h, 10c	GC-MS, DTD-GC-MS		7118	Dimethoxybenzenes
2-butenal	10c	GC-MS		447466	Enals
4,5-epoxydec-2(trans)-enal	10c	GC-O		15825667	Enals
(5Z)-octa-1,5-dien-3-one	10c	GC-O		6429343	Enones
1-octen-3-one	10c	GC-O	herbal, earthy, metal, musty, mushroom, dirty	61346	Enones
3-octen-2-one	10c	GC-MS, GC-O	crushed bug, nut, herbal, earthy, hay, sweet, blueberry, mushroom, spicy	5363229	Enones
4-hexen-3-one	10c	GC-MS	ethereal, pungent, tropical, metallic, spicy, green	5365811	Enones
ethyl caprate	10c	GC-MS	apple, brandy, waxy, grape, oily, sweet, fruity, pear	8048	Fatty acid esters
ethyl caprylate	10c	GC-MS	apricot, fat, wine, waxy, banana, brandy, fruit, sweet, fruity, pear	7799	Fatty acid esters
ethyl oleate	10c	GC-MS		5363269	Fatty acid esters

ethyl palmitate	8h, 10c	GC-MS	mild, wax, milky, waxy, creamy, fruity, balsam	12366	Fatty acid esters
isopropyl myristate	10c	GC-MS	oily, fatty, faint, cinnamon	8042	Fatty acid esters
methyl cis- l2-octadecenoate	10c	GC-MS		5370350	Fatty acid esters
methyl palmitate	15h, 10c	GC-MS	oily, fatty, waxy	8181	Fatty acid methyl esters
1-dodecanol	10c	GC-MS	coconut, wax, fat, waxy, fatty, earthy, honey, soapy	8193	Fatty alcohols
1-hexanol	10c	GC-MS	oil, alcoholic, ethereal, resin, fusel, sweet, fruity, flower, green	8103	Fatty alcohols
1-octen-3-ol	10c	GC-MS	raw, fishy, oily, earthy, fungal, chicken, mushroom, green	18827	Fatty alcohols
heptacosan-1-ol	8h, 15h	DTD-GC-MS		74822	Fatty alcohols
hexacosan-1ol	8h, 15h	DTD-GC-MS		68171	Fatty alcohols
octacosan-1-ol	15h	DTD-GC-MS		68406	Fatty alcohols
cis-9-octadecenal	10c	GC-MS		5364492	Fatty aldehydes
hexacosanal	8h	DTD-GC-MS		3084462	Fatty aldehydes
luteolin 7-glucoside-4'-(Z-2-methyl-2-butenoate)	1a, 2a	UHPLC-MS/MS	sweet-like	44258141	Flavonoid-7-O-glycosides
wyerol	1a, 2a	UHPLC-MS/MS		131752561	Furanoid fatty acids
2-hydroxyfuraneol	10c	GC-MS		538757	Furanones
furaneol	8h, 15h	DTD-GC-MS	caramel, strawberry, grape, cotton, sweet, sugar, candy, almond	19309	Furanones
methyl furan-2-carboxylate	8h	DTD-GC-MS	tobacco, fungal, sweet, fruity, mushroom	11902	Furoic acid esters

3-pentyl-5-methyldihydro-2(3H)-furanone	10c	GC-MS		12393246	Gamma butyrolactones
a,d-dimethyltetronic acid	8h	DTD-GC-MS		228435	Gamma butyrolactones
γ-butyrolactone	8h, 15h	DTD-GC-MS	caramel, oily, fatty, sweet, creamy	7302	Gamma butyrolactones
γ-nonalactone	10c	GC-O	peach, coconut, waxy, oily, creamy, sweet, buttery	7710	Gamma butyrolactones
4-oxopentanoic acid	8h	DTD-GC-MS	caramel, acetoin, acidic, sweet, buttery	11579	Gamma-keto acids and derivatives
galactosylglycerol	1a, 2a	UHPLC-MS/MS	sweet	16048618	Glycosylglycerols
2-methyl-3-furanthiol	10c	GC-O	coconut, meat, sulfury, fishy, metallic, meaty	34286	Heteroaromatic compounds
2-pentylfuran	10c	GC-MS	butter, green bean, vegetable, earthy, beany, fruity, metallic, green	19602	Heteroaromatic compounds
3-furfurol	10c	GC-MS		20449	Heteroaromatic compounds
furfuryl alcohol	8h, 15h	DTD-GC-MS	coffee, alcoholic, bitter, musty, chemical, caramel, sweet, burnt, bread	7361	Heteroaromatic compounds
glucose (D-glucose, α-glucose, β-glucose)	1a, 2a, 5d, 11d, 13d	NMR, UHPLC-MS/MS	sweet	5793	Hexoses
L-histidine	1a, 2a	UHPLC-MS/MS	bitter	6274	Histidine and derivatives
glucosyringic acid	1a, 2a	UHPLC-MS/MS		10383888	Hydrolyzable tannins
newbouldioside A	1a, 2a	UHPLC-MS/MS		102180527	Hydrolyzable tannins
vanillic acid 4-beta-D-glucoside	1a, 2a	UHPLC-MS/MS		14132336	Hydrolyzable tannins
citramalic acid	1a, 2a	UHPLC-MS/MS		1081	Hydroxy fatty acids
ethyl vanillin	10b	HPLC	vanilla, caramel, sweet, creamy	8467	Hydroxybenzaldehydes

p-hydroxybenzaldehyde	1ag, 2a, 6g, 7g, 8h, 10bc, 11eg, 12g, 14g, 15h	GC-MS, HPLC, HPLC-DAD, DTD-GC-MS, UHPLC-MS/MS	woody, sweet, balsam, nutty, almond	126	Hydroxybenzaldehydes
p-hydroxybenzoic acid	1ag, 2a, 3g, 4g, 6g, 7g, 9h, 10b, 11eg, 12g, 14g, 15h	HPLC, HPLC-DAD, DTD-GC-MS, UHPLC-MS/MS	nutty, phenolic	135	Hydroxybenzoic acid derivatives
protocatechuic acid	1a, 2a	UHPLC-MS/MS	phenolic, balsamic, mild	72	Hydroxybenzoic acid derivatives
1-O-sinapoylglucose	1a, 2a	UHPLC-MS/MS		6168296	Hydroxycinnamic acid glycosides
p-hydroxycinnamic acid	1a, 2a	UHPLC-MS/MS	balsam, balsamic	637542	Hydroxycinnamic acids
plumieride	1a, 2a	UHPLC-MS/MS		72319	Iridoid O-glycosides
tectoridin	1a, 2a	UHPLC-MS/MS	sweet-like	5281810	Isoflavonoid O-glycosides
isoleucine	5d, 11d, 13d	NMR		6306	Isoleucine and derivatives
acetoin propyleneglycol acetal	10c	GC-MS		11971259	Ketals
2-heptanone	10c	GC-MS	coconut, soap, herbal, sweet, woody, fruity, spicy, cinnamon	8051	Ketones
2-nonanone	10c	GC-MS	soap, herbal, fresh, fishy, hot milk, earthy, sweet, soapy, weedy, green	13187	Ketones
3-methylpentan-2-one	15h	DTD-GC-MS		11262	Ketones
3-octanone	10c	GC-MS	butter, herbal, resin, fresh, mushroom, sweet, lavender, herb	246728	Ketones
pentan-2-one	15h	DTD-GC-MS	potato, alcohol, ether, ethereal, wine, banana, fishy, fruit, sweet, woody, fruity	7895	Ketones
leucine	5d, 11d, 13d	NMR		6106	Leucine and derivatives
9,12-octadecadienoic acid	8h, 15h	DTD-GC-MS		3931	Lineolic acids and derivatives

ethyl linoleate	10c	GC-MS		5282184	Lineolic acids and derivatives
ethyl linolenate	10c	GC-MS		5367460	Lineolic acids and derivatives
linoleic acid	10c	GC-MS	fatty, faint	5280450	Lineolic acids and derivatives
heptadecanoic acid	8h, 15h	DTD-GC-MS		10465	Long-chain fatty acids
hexadecanoic acid	8h, 15h, 10c	GC-MS	fatty, slightly waxy	985	Long-chain fatty acids
octadecanoic acid	8h, 15h	DTD-GC-MS	fatty, odorless, mild	5281	Long-chain fatty acids
oleic acid	1a, 2a, 8h, 15h	DTD-GC-MS, UHPLC-MS/MS	lard, fat, waxy, fatty, fried, faint	445639	Long-chain fatty acids
palmitoleic acid	15h	DTD-GC-MS		445638	Long-chain fatty acids
pentadecanoic acid	8h, 15h, 10c	GC-MS	waxy	13849	Long-chain fatty acids
tetradecanoic acid	8h, 15h, 10c	GC-MS	fatty, soapy, waxy, coconut	11005	Long-chain fatty acids
1-hexadecanol	10c	GC-MS	wax, floral, waxy, flower	2682	Long-chain fatty alcohols
1-octadecanol	10c	GC-MS	oil, bland	8221	Long-chain fatty alcohols
(2E,4E)-deca-2,4-dienal	10c	GC-MS, GC-O	citrus, orange, nut, wax, meat, fat, fresh, fatty, oily, cucumber, sweet, melon, pumpkin, fried, green	5283349	Medium-chain aldehydes
(2E,4Z)-deca-2,4-dienal	10c	GC-MS, GC-O	fat, waxy, fatty, geranium, fried, green	6427087	Medium-chain aldehydes
(2E,6E)-nona-2,6-dienal	10c	GC-O	citrus, fresh, green, melon, cucumber	636687	Medium-chain aldehydes
10-undecenal	10c	GC-O	citrus, rose, waxy, mandarin, fatty, aldehydic, soapy	8187	Medium-chain aldehydes
2,4-nonadienal	10c	GC-O		5283339	Medium-chain aldehydes
2-undecenal	10c	GC-O	citrus, soap, orange peel, fat, fresh, sweet, fruity, green	5283356	Medium-chain aldehydes

3,3-dimethylhexanal	8h	DTD-GC-MS		558458	Medium-chain aldehydes
cis-3-hexenal	10c	GC-O	apple, fatty, fruity, leaf, grassy, weedy, green	643941	Medium-chain aldehydes
cis-3-nonenal	10c	GC-MS, GC-O	cucumber	6431042	Medium-chain aldehydes
decanal	8h, 15h	DTD-GC-MS	citrus, soap, orange peel, tallow, waxy, floral, sweet, aldehydic	8175	Medium-chain aldehydes
heptanal	10c	GC-MS	citrus, ozone, fat, herbal, fresh, wine-lee, rancid, fatty, aldehydic, green	8130	Medium-chain aldehydes
hexanal	10c	GC-O	leafy, grass, sweaty, tallow, fat, fresh, fatty, fruity, aldehydic, green	6184	Medium-chain aldehydes
nonanal	8h, 15h, 10c	GC-MS, GC-O	citrus, lime, orange peel, rose, fat, green, fishy, waxy, fresh, fatty, peely, aldehydic, orris, grapefruit	31289	Medium-chain aldehydes
octanal	10c	GC-O	lemon, citrus, soap, orange peel, fat, waxy, fatty, aldehydic, green	454	Medium-chain aldehydes
trans-2-heptenal	10c	GC-MS, GC-O	soap, vegetable, fat, fresh, fatty, pungent, almond, green	5283316	Medium-chain aldehydes
trans-2-nonenal	10c	GC-MS	waxy, green, fatty, paper, melon, cucumber	5283335	Medium-chain aldehydes
trans-2-octenal	10c	GC-MS	nut, fat, herbal, fresh, green, fatty, banana, waxy, leaf, cucumber	5283324	Medium-chain aldehydes
decanoic acid	10c	GC-MS	sour, citrus, fat, rancid, fatty, unpleasant	2969	Medium-chain fatty acids
heptanoic acid	10c	GC-MS	sour, cheesy, sweat, rancid, cheese	8094	Medium-chain fatty acids
nonanoic acid	10c	GC-MS	cultured dairy, fat, waxy, green, dirty, cheese	8158	Medium-chain fatty acids

carvone	16i	GC-MS		7439	Menthane monoterpenoids
cis-beta-terpineol	16i	GC-MS		8748	Menthane monoterpenoids
cis-carveol	16i	GC-MS		330573	Menthane monoterpenoids
L-h-terpineol	16i	GC-MS		17100	Menthane monoterpenoids
limonene	10c	GC-MS	lemon, citrus, orange, herbal, woody, terpene, citric, camphor	22311	Menthane monoterpenoids
S-(-)-limonene	16i	GC-MS		439250	Menthane monoterpenoids
terpinolene	16i	GC-MS		11463	Menthane monoterpenoids
trans-dihydrocarvone	16i	GC-MS		6432474	Menthane monoterpenoids
α-terpineol	9h	DTD-GC-MS	oil, mint, citrus, lilac, floral, woody, anise, terpene, pine	442501	Menthane monoterpenoids
4-hydroxy-3- methoxyphenylacetone	10c	GC-MS		17262	Methoxyphenols
4-vinylguaiacol	1a, 2a, 8h, 10c	GC-MS, UHPLC-MS/MS, GC-O	curry, smoky, clove, peanut, spicy	332	Methoxyphenols
creosol	8h, 10c, 15h	DTD-GC-MS, GC-MS, GC-O	phenolic, clove, leather, smoky, bacon, medical, mesquite, carnation, medicinal, bitter, vanilla, spice	7144	Methoxyphenols
guaiacol	8h, 15h, 10c	GC-MS	spice, vanilla, smoky, sweet, woody, phenolic, medicine, medicinal, smoke	460	Methoxyphenols
homovanillic acid	10c	GC-MS		1738	Methoxyphenols
syringic aldehyde	15h	DTD-GC-MS	mild, sweet, woody, tonka, plastic	8655	Methoxyphenols
vanillin	1a, 2a, 6g, 7g, 8h, 9h, 10bc, 11eg, 14g, 15h	GC-MS, HPLC, HPLC-DAD, UHPLC-MS/MS, GC-O	vanilla, chocolate, sweet, creamy	1183	Methoxyphenols

vanillin 1,2-glyceryl acetal I	10c	GC-MS		82115125	Methoxyphenols
vanillyl acetone	15h	DTD-GC-MS	vanilla, clove, animal, sweet, ginger, woody, spicy, phenolic	31211	Methoxyphenols
vanillyl alcohol	1ag, 2a, 3g, 6g, 7g, 9h, 11eg, 12g, 14g, 15h	HPLC, HPLC-DAD, DTD-GC-MS, UHPLC-MS/MS	vanilla, sweet, anise, creamy, balsam, tonka, phenolic	62348	Methoxyphenols
methyl acetate	10c	GC-MS	sweet, bitter, ether, fruity	6584	Methyl esters
2-methylbutyric acid	10c	GC-MS	sour, sweat, acid, strawberry, roquefort cheese, pungent, cheese	8314	Methyl-branched fatty acids
isovaleric acid	10c	GC-MS, GC-O	sour, sweat, acid, stinky, sweaty, animal, rancid, tropical, feet, cheese	10430	Methyl-branched fatty acids
methyl vanillate	8h, 15h, 10c	GC-MS	butterscotch, caramel, warm, vanilla, spicy	19844	M-methoxybenzoic acids and derivatives
vanillic acid	1ag, 2a, 6g, 7g, 8h, 9h, 10b, 11eg, 12g, 15h	HPLC, HPLC-DAD, DTD-GC-MS, NMR, UHPLC-MS/MS	powdery, vanilla, bean, milky, sweet, creamy, dairy	8468	M-methoxybenzoic acids and derivatives
1-hydroxy-2-pentanone	10c	GC-MS		522131	Monosaccharides
fructose (α-fructose, β-fructose)	5d, 11d, 13d	NMR		5984	Monosaccharides
1,4-dimethylpiperazine	15h	DTD-GC-MS		7818	N-methylpiperazines
6-O-alpha-L-rhamnopyranosylcatalpol	1a, 2a	UHPLC-MS/MS		102252680	O-glycosyl compounds
rhodioloside	1a, 2a	UHPLC-MS/MS		159278	O-glycosyl compounds
sucrose	5d, 11d, 13d	NMR		5988	O-glycosyl compounds
verbasoside	1a, 2a	UHPLC-MS/MS		11754080	O-glycosyl compounds
anisyl salicylate	10c	GC-MS		3085862	O-Hydroxybenzoic acid esters
trans-carvone oxide	16i	GC-MS		11829800	Oxepanes

cis-carvone oxide	16i	GC-MS		11030188	Oxepanes
limonene oxide	16i	GC-MS		91496	Oxepanes
limonene oxide, cis	16i	GC-MS		6432449	Oxepanes
limonene oxide, trans	16i	GC-MS		8029780	Oxepanes
D-arabinose	1a, 2a	UHPLC-MS/MS	sweet	439195	Pentoses
methyl creosol	10c	GC-MS		75715	Phenol ethers
apiopaeonoside	1a, 2a	UHPLC-MS/MS		127509	Phenolic glycosides
arbutin	1a, 2a	UHPLC-MS/MS	bitter	440936	Phenolic glycosides
coelovirin A or B	1a, 2a	UHPLC-MS/MS		132472119	Phenolic glycosides
glucovanillin	1a, 2a	UHPLC-MS/MS	bitter	6452133	Phenolic glycosides
icariside D2	1a, 2a	UHPLC-MS/MS		10614148	Phenolic glycosides
paeonoside	1a, 2a	UHPLC-MS/MS		442924	Phenolic glycosides
pseudolaroside A	1a, 2a	UHPLC-MS/MS			Phenolic glycosides
phenylacetaldehyde	1a, 2a	UHPLC-MS/MS	hyacinth, honey, clover, sweet, hawthorne, cocoa, grapefruit, green, peanut, floral, bitter	998	Phenylacetaldehydes
phenylalanine	1a, 2a, 5d, 11d, 13d	NMR, UHPLC-MS/MS	bitter (L), sweet (D)	6140	Phenylalanine and derivatives
1-(4-methoxyphenyl)- 2-methyl-3-buten-1-ol	15h	DTD-GC-MS		586295	Phenylpropanes
propyl 4-hydroxybenzoate	8h	DTD-GC-MS	burnt, smoky, sweet, woody, hawthorn	7175	*p*-Hydroxybenzoic acid alkyl esters
isovanillic acid	10c	GC-MS		12575	*p*-methoxybenzoic acids and derivatives
p-anisic acid	8h, 10bc, 15h	GC-MS, HPLC, HPLC-DAD, DTD-GC-MS	putrid, sweet, faint, cadaverous	7478	*p*-methoxybenzoic acids and derivatives

1-butanol	10c	GC-MS	oil, vanilla, fruit, fusel, sweet, balsam, medicine, medicinal	263	Primary alcohols
1-pentanol	10c	GC-MS	oil, balsamic, vanilla, fusel, sweet, balsam	6276	Primary alcohols
ethanol	5d, 11d, 13d	NMR		702	Primary alcohols
neoheptanol	8h	DTD-GC-MS		16911	Primary alcohols
maltol	8h, 15h, 10c	GC-MS, GC-O	caramel, jam, bread, cotton, sweet, fruity, baked, candy	8369	Pyranones and derivatives
2-ethylpyrazine	10c	GC-MS	butter, peanut, musty, cocoa, nutty, wood, roasted, woody, bitter, peanut butter	26331	Pyrazines
2-acetyl-1-pyrroline	10c	GC-O	roast, nut, roasted, ham, sweet, nutty	522834	Pyrrolines
quinic acid	1a, 2a	UHPLC-MS/MS		6508	Quinic acids and derivatives
hexan-2-ol	15h	DTD-GC-MS	chemical, winey	12297	Secondary alcohols
trans-3-penten-2-ol	10c	GC-MS	vinyl, green	5366239	Secondary alcohols
alpha-cadinol	10c	GC-MS		10398656	Sesquiterpenoids
alpha-calacorene	10c	GC-MS		12302243	Sesquiterpenoids
alpha-d-curcumene	10c	GC-MS	herb	92139	Sesquiterpenoids
cadalene	10c	GC-MS		10225	Sesquiterpenoids
calamenene	10c	GC-MS		6429077	Sesquiterpenoids
delta-cadinene	10c	GC-MS	herbal, woody, thyme, wood, medicine, dry	441005	Sesquiterpenoids
epi-alpha-cadinol	10c	GC-MS	earthy, odorless, balsam	160799	Sesquiterpenoids
epi-alpha-muurolol	10c	GC-MS	spicy, herbal, weak spice, herb	3084331	Sesquiterpenoids

gamma-muurolene	10c	GC-MS		12313020	Sesquiterpenoids
hexahydrofarnesyl acetone	10c	GC-MS	jasmin, herbal, fat, oily, woody, celery	10408	Sesquiterpenoids
acetaldehyde	15h	DTD-GC-MS	ethereal, ether, whiskey, pungent, fruity, aldehydic	177	Short-chain aldehydes
4-methyl-2 oxovaleric acid	15h	DTD-GC-MS	fruity	70	Short-chain keto acids and derivatives
butanoic acid	10c	GC-MS	butter, sweat, acetic, sharp, rancid, fruit, cheese	264	Straight chain fatty acids
4-vinylphenol	1a, 2a, 10c	GC-MS, UHPLC-MS/MS	medical, chemical, almond shell, sweet, phenolic	62453	Styrenes
styrene	10c	GC-MS	balsamic, gasoline, floral, sweet, balsam, plastic	7501	Styrenes
2-methyl-3-buten-2-ol	10c	GC-MS	oily, earthy, herbal, herb	8257	Tertiary alcohols
tert-amyl alcohol	10c	GC-MS	pungent	6405	Tertiary alcohols
homocitric acid (in its lactone form)	5d, 13d	NMR		28371	Tricarboxylic acids and derivatives
squalene	8h, 15h, 10c	GC-MS,		638072	Triterpenoids
tyrosine	5d, 13d	NMR	sweet (D)	6057	Tyrosine and derivatives
tyrosol	1a, 2a	UHPLC-MS/MS	floral, sweet, fruity, mild	10393	Tyrosols
1-eicosene	10c	GC-MS		18936	Unsaturated aliphatic hydrocarbons
1-tricosene	8h, 15h	DTD-GC-MS		181154	Unsaturated aliphatic hydrocarbons
cis-9-tricosene	10c	GC-MS		5365075	Unsaturated aliphatic hydrocarbons

docosene	15h	DTD-GC-MS	74138	Unsaturated aliphatic hydrocarbons
hexacosene	15h	DTD-GC-MS	29303	Unsaturated aliphatic hydrocarbons
nonacos-1-ene	8h, 15h	DTD-GC-MS	156989	Unsaturated aliphatic hydrocarbons
pentacos-1-ene	15h	DTD-GC-MS	528972	Unsaturated aliphatic hydrocarbons
valine	5d, 11d, 13d	NMR	6287	Valine and derivatives
5,7-dihydroxy-2-(3-hydroxy-4-methoxy-phenyl)-3-methoxy-chromone	1a, 2a	UHPLC-MS/MS		
5-vinyl-guaiacol	10c	GC-O		
anisyl palmitate	8h, 15h, 10c	GC-MS		
dracunculifoside J	1a, 2a	UHPLC-MS/MS		
glucoside A	11d	NMR		
glucoside B	5d, 11d, 13d	NMR		
hydroxydi-hydromaltol	8h, 15h	DTD-GC-MS		
methyl-2-(4-hydroyphenoxy) benzoate	15h	DTD-GC-MS		
p-hydroxybenzyl alcohol glucoside	5d, 11d, 13d	NMR		
γ-aminobutiric acid	5d, 11d, 13d	NMR		

1 = *V. bahiana*, 2 = *V. chamissonis*, 3 = *V. crenulata*, 4 = *V. imperialis*, 5 = *V. palmarum*, 6 = *V. planifolia* x *V. phaeantha*, 7 = *V. planifolia* x *V. pompona*, 8 = *V. pompona* (from Madagascar), 9 = *V. pompona* (origin unknown), 10 = *V. pompona* Shiede (cured), 11 = *V. pompona* subsp *grandiflora*, 12 = *V. pompona* subsp *pittieri*, 13 = *V. ribeiroi*, 14 = *V. sotoarenasii*, 15 = Wild Type (from Peru), 16 = *V. pompona* (flowers); a = [1], b = [2], c = [3], d = [4] *, e = [5], f = [6] *, g = [7], h = [8], i = [9] **. * Study based on leaves. ** Study based on flowers.

References

1. da Silva Oliveira, Joana Paula, Rafael Garrett, Maria Gabriela Bello Koblitz, and Andrea Furtado Macedo. "Vanilla flavor: Species from the Atlantic forest as natural alternatives." *Food Chemistry* 375 (2022): 131891. https://doi.org/10.1016/j.foodchem.2021.131891
2. Ehlers, Dorothea, and Michael Pfister. "Compounds of vanillons (Vanilla pompona Schiede)." *Journal of Essential Oil Research* 9, no. 4 (1997): 427-431. https://doi.org/10.1080/10412905.1997.9700743
3. Galeas, Maria Del Pilar. "Gas chromatography-mass spectrometry and gas chromatography-olfactometry analysis of aroma compounds of Vanilla pompona Schiede." PhD diss., Rutgers The State University of New Jersey-New Brunswick, 2015.
4. Leyva, Vanessa E., Juan M. Lopez, Alvaro Zevallos-Ventura, Rodrigo Cabrera, Cristhian Cañari-Chumpitaz, David Toubiana, and Helena Maruenda. "NMR-based leaf metabolic profiling of V. planifolia and three endemic Vanilla species from the Peruvian Amazon." *Food Chemistry* 358 (2021): 129365. https://doi.org/10.1016/j.foodchem.2021.129365
5. Maruenda, Helena, Maria del Lujan Vico, J. Ethan Householder, John P. Janovec, Cristhian Cañari, Angelica Naka, and Ana E. Gonzalez. "Exploration of Vanilla pompona from the Peruvian Amazon as a potential source of vanilla essence: Quantification of phenolics by HPLC-DAD." *Food Chemistry* 138, no. 1 (2013): 161-167. https://doi.org/10.1016/j.foodchem.2012.10.037
6. Palama, Tony Lionel, Michel Grisoni, Isabelle Fock-Bastide, Katia Jade, Laetitia Bartet, Young Hae Choi, Robert Verpoorte, and Hippolyte Kodja. "Metabolome of Vanilla planifolia (Orchidaceae) and related species under Cymbidium mosaic virus (CymMV) infection." *Plant physiology and biochemistry* 60 (2012): 25-34. https://doi.org/10.1016/j.plaphy.2012.07.015
7. Pérez-Silva, Araceli, Mayra Nicolás-García, Thomas Petit, Jean Bernard Dijoux, María de los Ángeles Vivar-Vera, Pascale Besse, and Michel Grisoni. "Quantification of the aromatic potential of ripe fruit of Vanilla planifolia (Orchidaceae) and several of its closely and distantly related species and hybrids." *European Food Research and Technology* 247, no. 6 (2021): 1489-1499. https://doi.org/10.1007/s00217-021-03726-w
8. Toth, Stephen, Keun Joong Lee, Daphna Havkin-Frenkel, Faith C. Belanger, and Thomas G. Hartman, eds. "Volatile compounds in vanilla." *Handbook of vanilla science and technology* (2018): 285-347. https://doi.org/10.1002/9781119377320.ch17
9. Watteyn, Charlotte, Daniela Scaccabarozzi, Bart Muys, Nele Van Der Schueren, Koenraad Van Meerbeek, Maria F. Guizar Amador, James D. Ackerman et al. "Trick or treat? Pollinator attraction in Vanilla pompona (Orchidaceae)." *Biotropica* 54, no. 1 (2022): 268-274.
